# Supplementary material for: Polyoxypregnane Glycosides from Root of Marsdenia tenacissima and Inhibited Nitric Oxide Levels in LPS Stimulated RAW 264.7 Cells
Source: Molecules. 2023 Jan 16;28(2):886. doi: 10.3390/molecules28020886 (PMC9861828; doi:10.3390/molecules28020886)
Supplement: Supplementary file 1 [file molecules-28-00886-s001.zip › molecules-2115637-supplementary.pdf]

Supplementary Material for:

Polyoxypregnane Glycosides from Root of *Marsdenia tenacissima* and Inhibited Nitric Oxide Levels in LPS Stimulated RAW 264.7 cells

Zhi Na [1,*], PianChou Gongpan [1], Qingfei Fan [2,*]

1 Key Laboratory of Tropical Plant Resources and Sustainable Use, Xishuangbanna Tropical Botanical Garden, Chinese Academy of Sciences, Mengla 666303, China; gongpan@xtbg.ac.cn (P.G.)

2 College of Science, Yunnan Agricultural University, Kunming 650201, China

* Correspondence: nazhi@xtbg.org.cn (Z. N.), fanqingfei@xtbg.ac.cn (Q.F.)

Contents

Figures

Figure S1. HRESIMS spectrum of compound **1**………. ……………………………… 4

Figure S2. ^{1}H-NMR (500 MHz, $CDCl_3$) spectrum of compound **1** ……………………5

Figure S3. ^{13}C-NMR and DEPT (125 MHz, $CDCl_3$) spectrum of compound **1**…………5

Figure S4. ^{1}H-^{1}H COSY spectrum of compound **1** ……………………………………...6

Figure S5. HSQC spectrum of compound **1**……………………………………………..6

Figure S6. HMBC spectrum of compound **1**……………………………………………7

Figure S7. ROESY spectrum of compound **1** …………………………………………..7

Figure S8. HREIMS spectrum of compound **2**………………………………… …….8

Figure S9. ^{1}H-NMR (500 MHz, $CDCl_3$) spectrum of compound **2** …………………......9

Figure S10. ^{13}C-NMR and DEPT (125 MHz, $CDCl_3$) spectrum of compound **2**…..........9

Figure S11. ^{1}H-^{1}H COSY spectrum of compound **2**……………………………………10

Figure S12. HSQC spectrum of compound **2**…………………………………………..10

Figure S13. HMBC spectrum of compound **2**…………………………………….......11

Figure S14. ROESY spectrum of compound **2**………………………………………....11

Figure S15. HRESIMS spectrum of compound **3**……………………………………....12

Figure S16. ^{1}H-NMR (500 MHz, $CDCl_3$) spectrum of compound **3**…………….…….13

Figure S17. ^{13}C-NMR and DEPT (125 MHz, $CDCl_3$) spectrum of compound **3**……,,..13

Figure S18. ^{1}H-^{1}H COSY spectrum of compound **3**…………………………………....14

Figure S19. HSQC spectrum of compound **3**……………………………………….....14

Figure S20. HMBC Spectrum of compound **3**…………………………………………….15

Figure S21. ROESY Spectrum of compound **3**…………………………………………15

Figure S22. HRESIMS spectrum of compound **4** ……………………………………...…16

Figure S23. ^{1}H-NMR (500 MHz, pyridine-d_5) spectrum of compound **4**………………17

Figure S24. ^{13}C-NMR and DEPT (125 MHz, pyridine-d_5) spectrum of compound **4**….17

Figure S25. ^{1}H-^{1}H COSY spectrum of compound **4**……………………………………18

Figure S26. HSQC spectrum of compound **4**……………………………………..……18

Figure S27. HMBC spectrum of compound **4**………………..…………………….....19

Figure S28. ROESY spectrum of compound **4**…………………………………………19

Figure S29. HRESIMS spectrum of compound **5**………………………………………20

Figure S30. ^{1}H-NMR (500 MHz, pyridine-d_5) spectrum of compound **5**………………21

Figure S31. ^{13}C-NMR and DEPT (125 MHz, pyridine-d_5) spectrum of compound **5**….21

Figure S32. ^{1}H-^{1}H COSY spectrum of compound **5**……………………………………22

Figure S33. HSQC spectrum of compound **5**…………………………………………...22

Figure S34. HMBC spectrum of compound **5**………………………………………….23

Figure S35. ROESY spectrum of compound **5**…………………………………………23

Figure S36. HRESIMS spectrum of compound **6**………………………………………24

Figure S37. ^{1}H-NMR (500 MHz, pyridine-d_5) spectrum of compound **6**………………25

Figure S38. ^{13}C-NMR and DEPT (125 MHz, pyridine-d_5) spectrum of compound **6**….25

Figure S39. ^{1}H-^{1}H COSY spectrum of compound **6**……………………………………26

Figure S40. HSQC spectrum of compound **6**…………………………………………...26

Figure S41. HMBC spectrum of compound **6**…………………………………………..27

Figure S42. ROESY spectrum of compound **6**…………………………………………27

Figure S43. HRESIMS spectrum of compound **7**………………………………………28

Figure S44. ^{1}H-NMR (500 MHz, pyridine-d_5) spectrum of compound **7**………………29

Figure S45. ^{13}C-NMR and DEPT (125 MHz, pyridine-d_5) spectrum of compound **7**….29

Figure S46. The ^{1}H-^{1}H COSY spectrum of compound **7**…………………..…………..30

Figure S47. HSQC spectrum of compound **7**…………………………………………...30

Figure S48. HMBC spectrum of compound **7**……………………………………….„31

Figure S49. ROESY spectrum of compound **7**…………………………………………31

Figure S50. Effects of compounds **1**–**7** on cell viability………………………………..32

Qualitative Analysis Report

**Data Filename**	ndbj-53.d	**Sample Name**	ndbj-53
**Sample Type**	Sample	**Position**	P1-F2
**Instrument Name**	Instrument 1	**User Name**	
**Acq Method**	s.m	**Acquired Time**	10/28/2020 2:27:28 PM
**IRM Calibration Status**	Success	**DA Method**	Default.m
**Comment**			

Sample Group **Info.**
Acquisition SW 6200 series TOF/6500 series
Version Q-TOF B.05.01 (B5125.2)

User Spectra

Fragmentor Voltage	Collision Energy	Ionization Mode
80	0	ESI

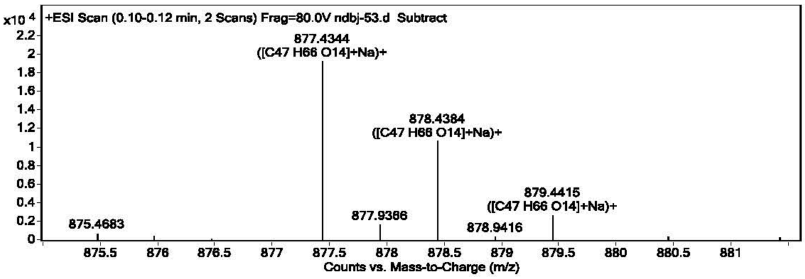

Peak List

m/z	z	Abund	Formula	Ion
551.301	1	1890.17		
673.2979	1	2904.21		
872.4793	1	3755.74		
877.4344	1	19341.66	C47 H66 O14	(M+Na)+
878.4384	1	10693.28	C47 H66 O14	(M+Na)+
879.4415	1	2738.25	C47 H66 O14	(M+Na)+
893.4087	1	14138.9		
894.4116	1	6676		
895.4125	1	3179.64		
1019.5192	1	3404.45		

Formula Calculator Element Limits

Element	Min	Max
C	3	120
H	0	240
O	0	30

Formula Calculator Results

Formula	CalculatedMass	CalculatedMz	Mz	Diff. (mDa)	Diff. (ppm)	DBE
C47 H66 O14	854.4453	877.4345	877.4344	0.10	0.11	15.0000

--- End Of Report ---

Figure S1 HRESIMS spectrum of compound **1**

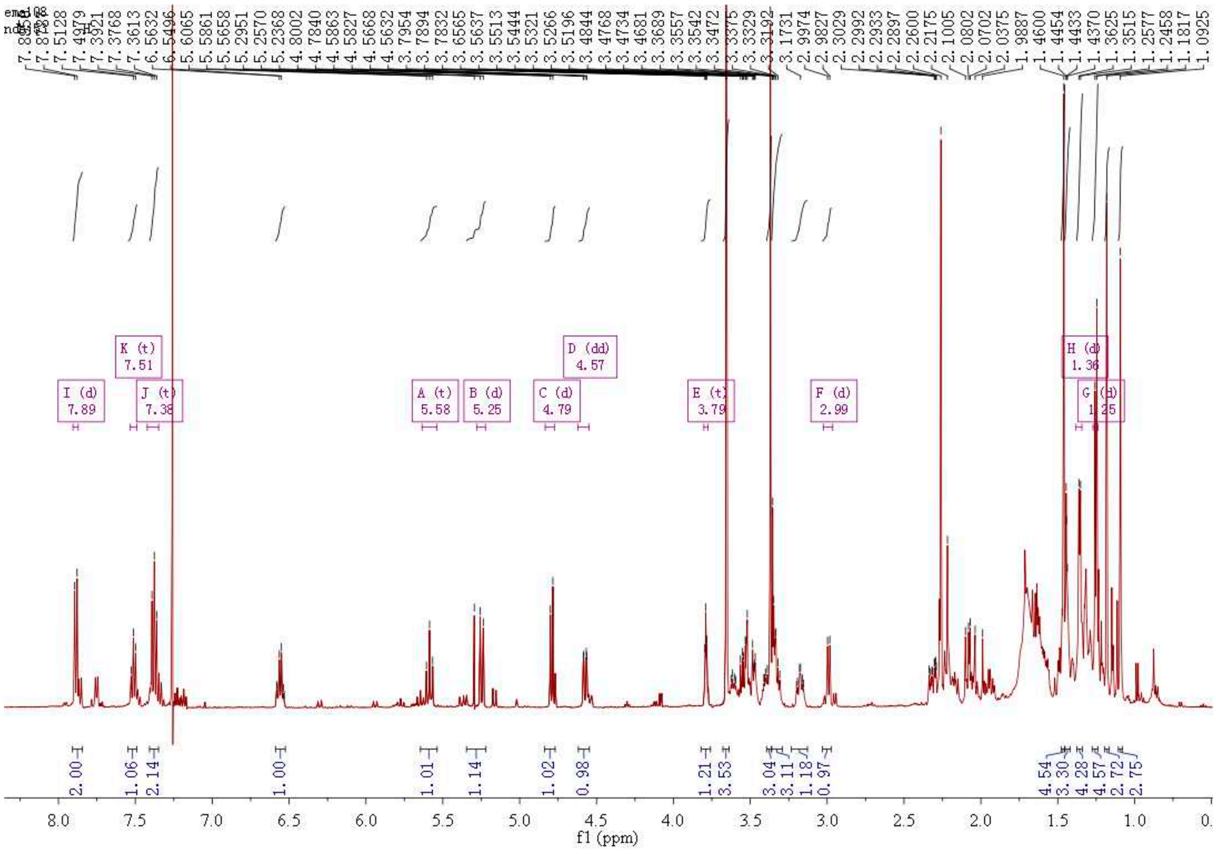

Figure S2 ^{1}H-NMR (500 MHz, $CDCl_3$) spectrum of compound **1**

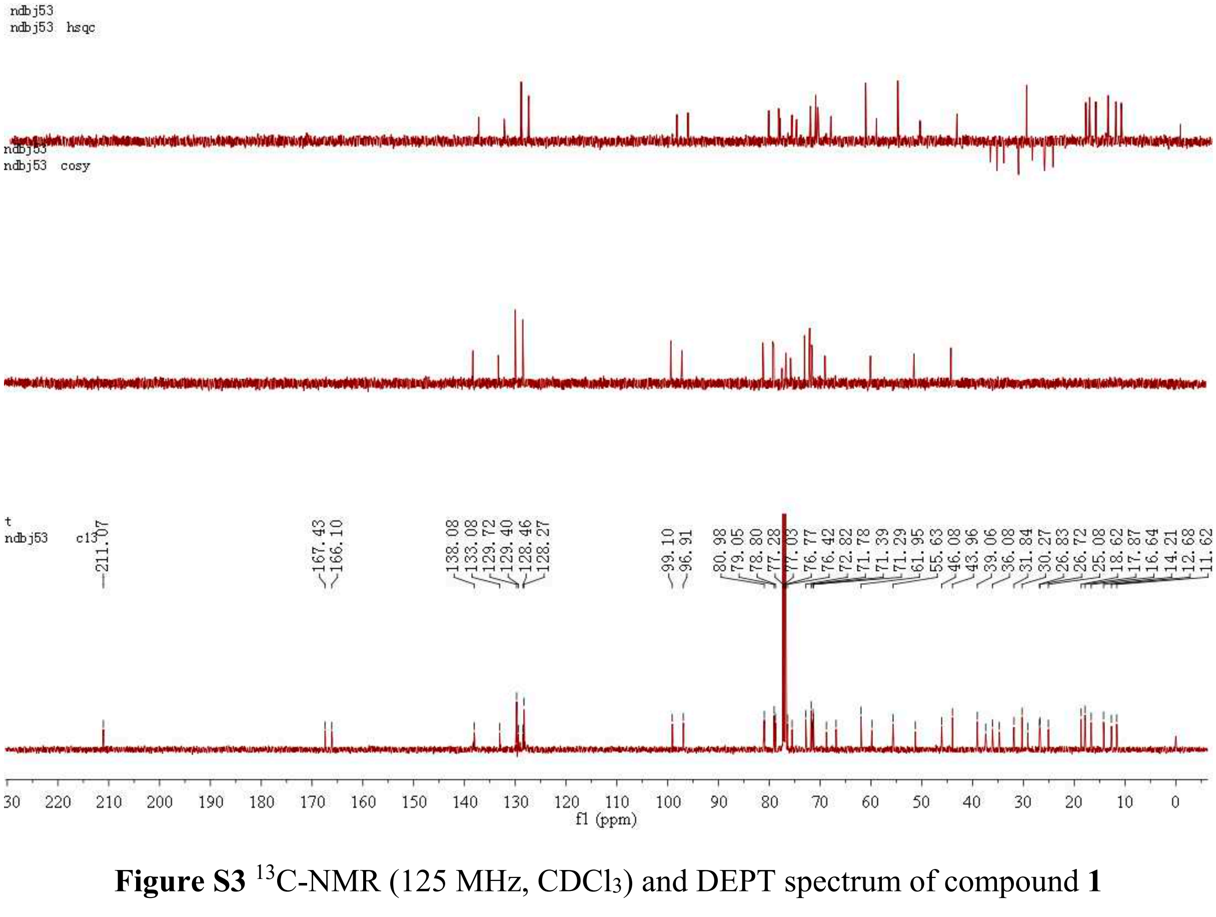

Figure S3 ^{13}C-NMR (125 MHz, $CDCl_3$) and DEPT spectrum of compound **1**

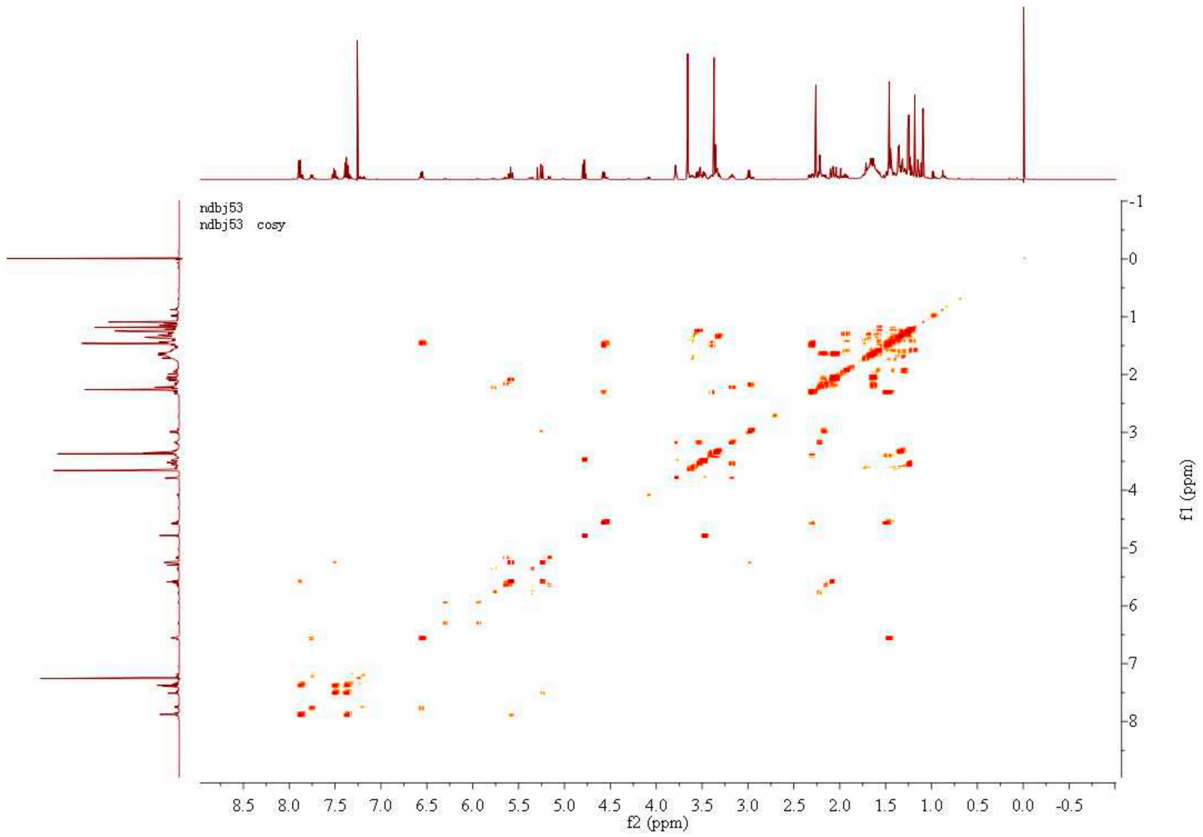

Figure S5 ^{1}H-^{1}H COSY spectrum of compound **1**

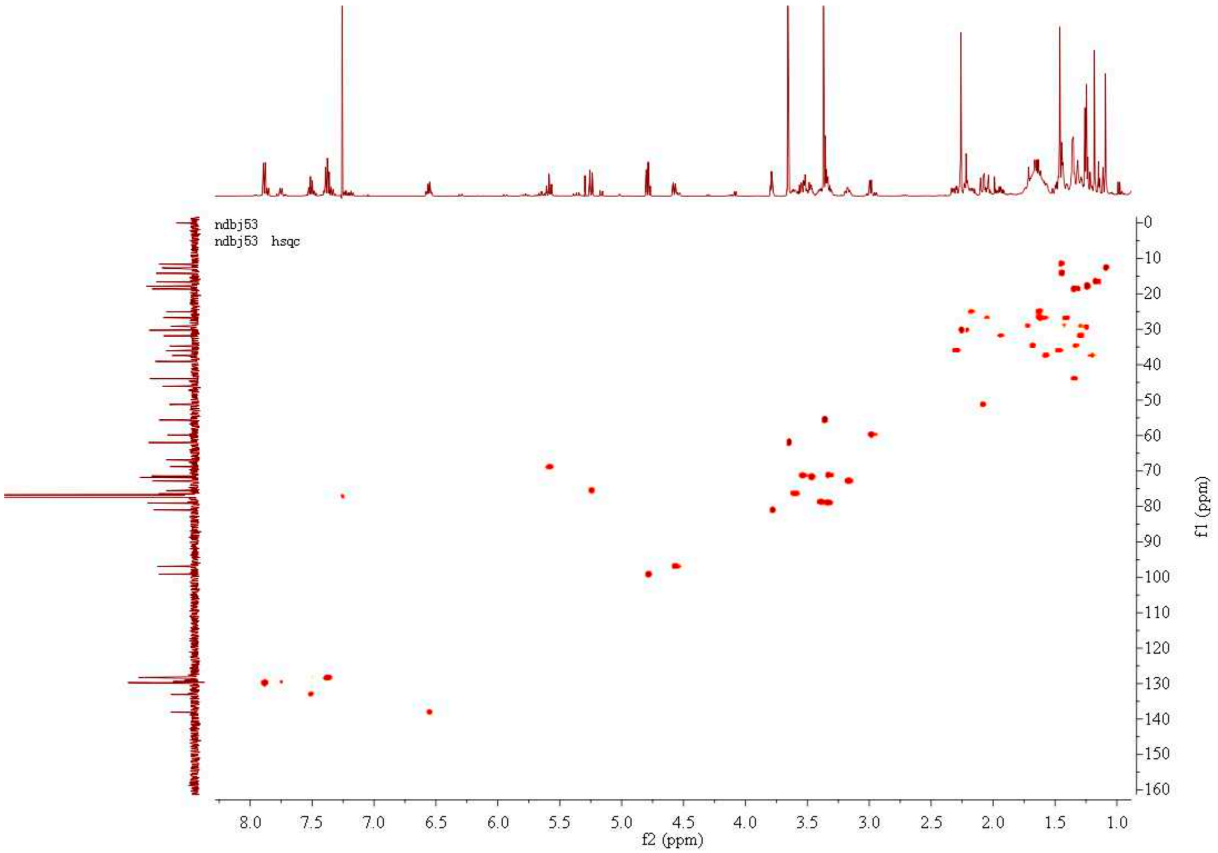

Figure S5 HSQC spectrum of compound **1**

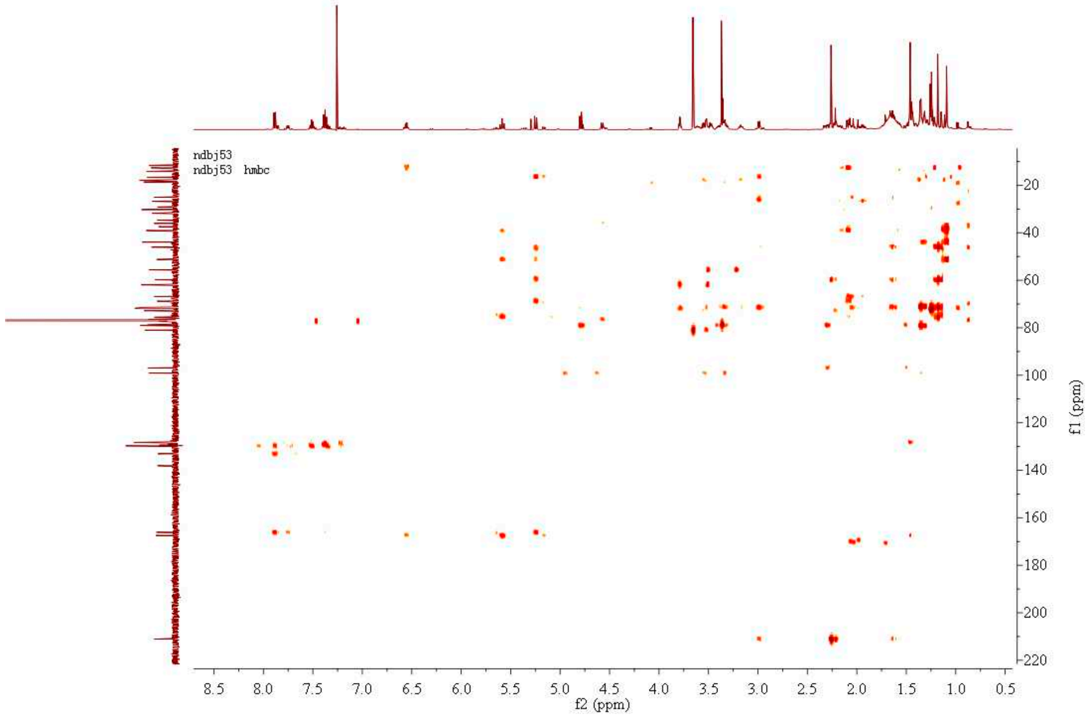

Figure S6 HMBC spectrum of compound **1**

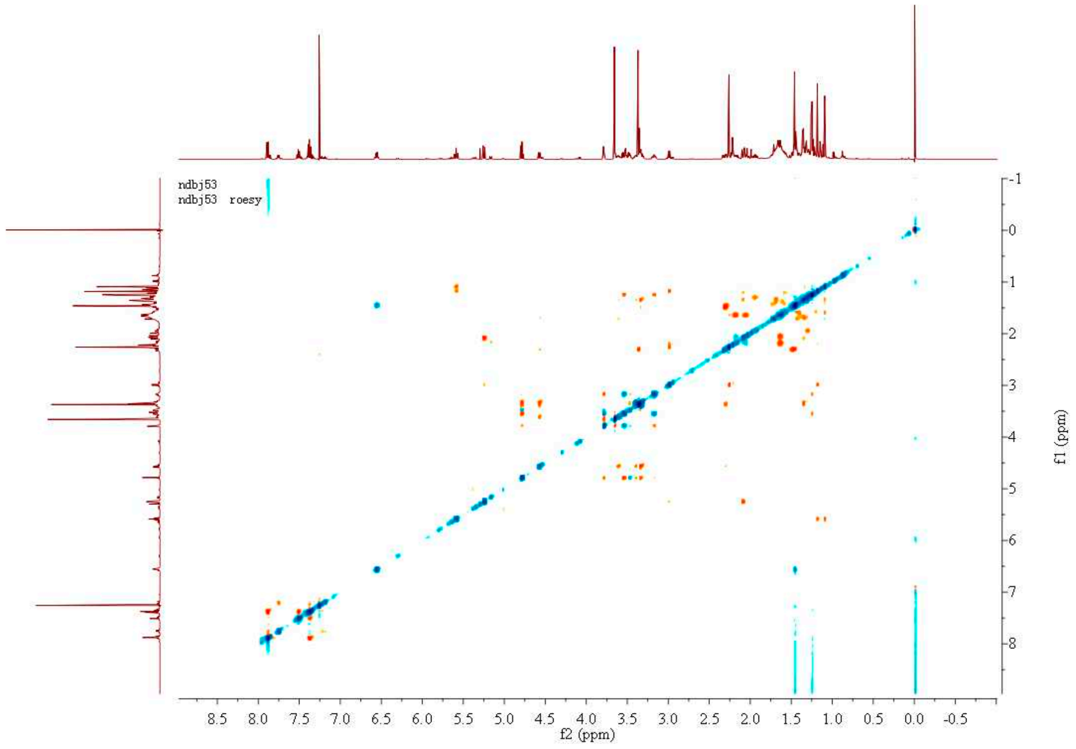

Figure S7 ROESY Spectrum of Compound **1**

Qualitative Analysis Report

**Data Filename**	ndbj-55.d	**Sample Name**	ndbj-55
**Sample Type**	Sample	**Position**	P1-F3
**Instrument Name**	Instrument 1	**User Name**	
**Acq Method**	s.m	**Acquired Time**	10/28/2020 2:28:39 PM
**IRM Calibration Status**	Success	**DA Method**	Default.m
**Comment**			

Sample Group **Info.**
Acquisition SW 6200 series TOF/6500 series
Version Q-TOF B.05.01 (B5125.2)

User Spectra

Fragmentor Voltage	Collision Energy	Ionization Mode
80	0	ESI

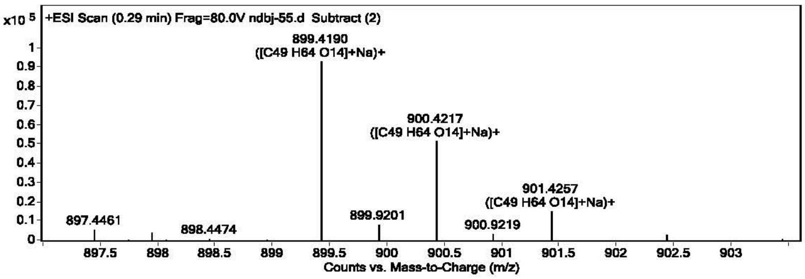

Peak List

m/z	z	Abund	Formula	Ion
377.2662	1	27443.59		
409.2559	1	15374.56		
673.298	1	10130.33		
865.4335	1	18937.1		
894.4545	1	11253.08		
899.419	1	93438.41	C49 H64 O14	(M+Na)+
900.4217	1	51815.04	C49 H64 O14	(M+Na)+
901.4257	1	15371.9	C49 H64 O14	(M+Na)+
915.3934	1	14416.72		
916.3973	1	9985.13		

Formula Calculator Element Limits

Element	Min	Max
C	3	120
H	0	240
O	0	30

Formula Calculator Results

Formula	CalculatedMass	CalculatedMz	Mz	Diff. (mDa)	Diff. (ppm)	DBE
C49 H64 O14	876.4296	899.4188	899.4190	-0.20	-0.22	18.0000

--- End Of Report ---

Figure S8 HRESIMS spectrum of compound **2**

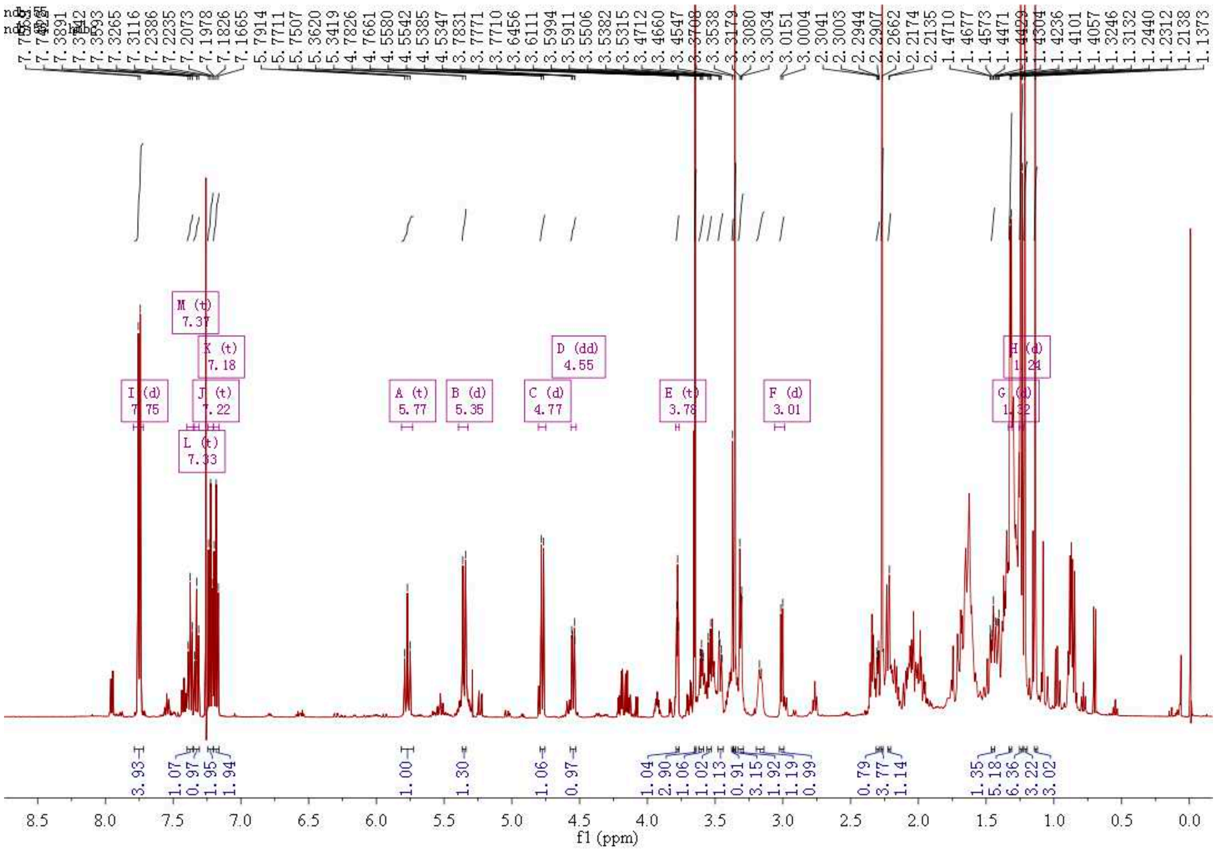

Figure S9 ^{1}H-NMR (500 MHz, $CDCl_3$) spectrum of compound **2**

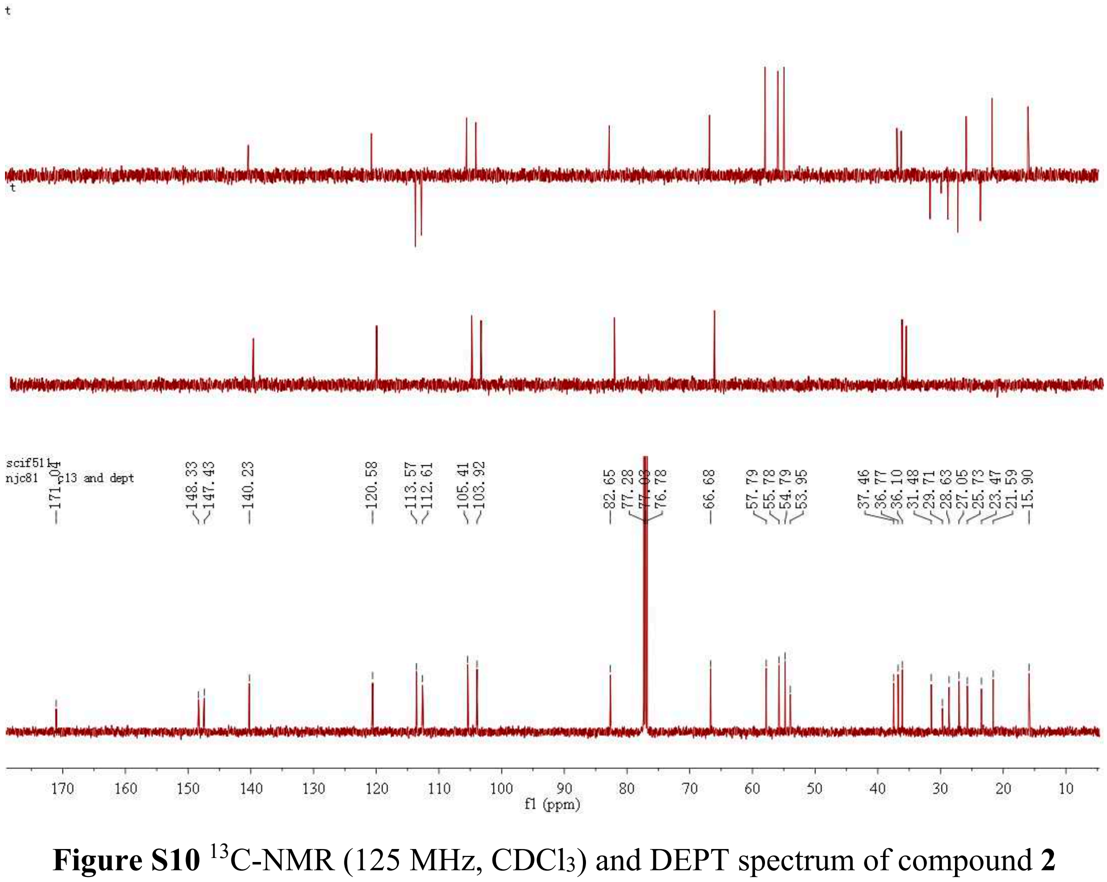

Figure S10 ^{13}C-NMR (125 MHz, $CDCl_3$) and DEPT spectrum of compound **2**

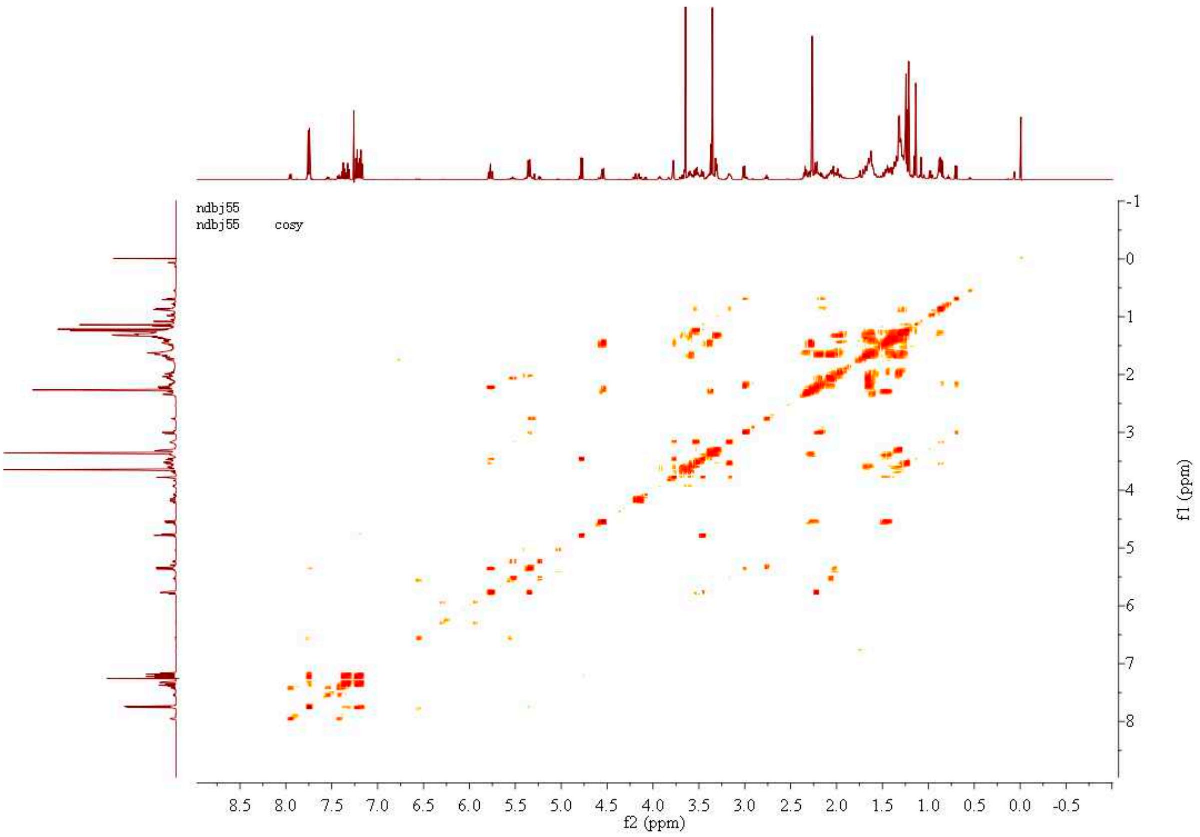

Figure S11 ^{1}H-^{1}H COSY spectrum of compound **2**

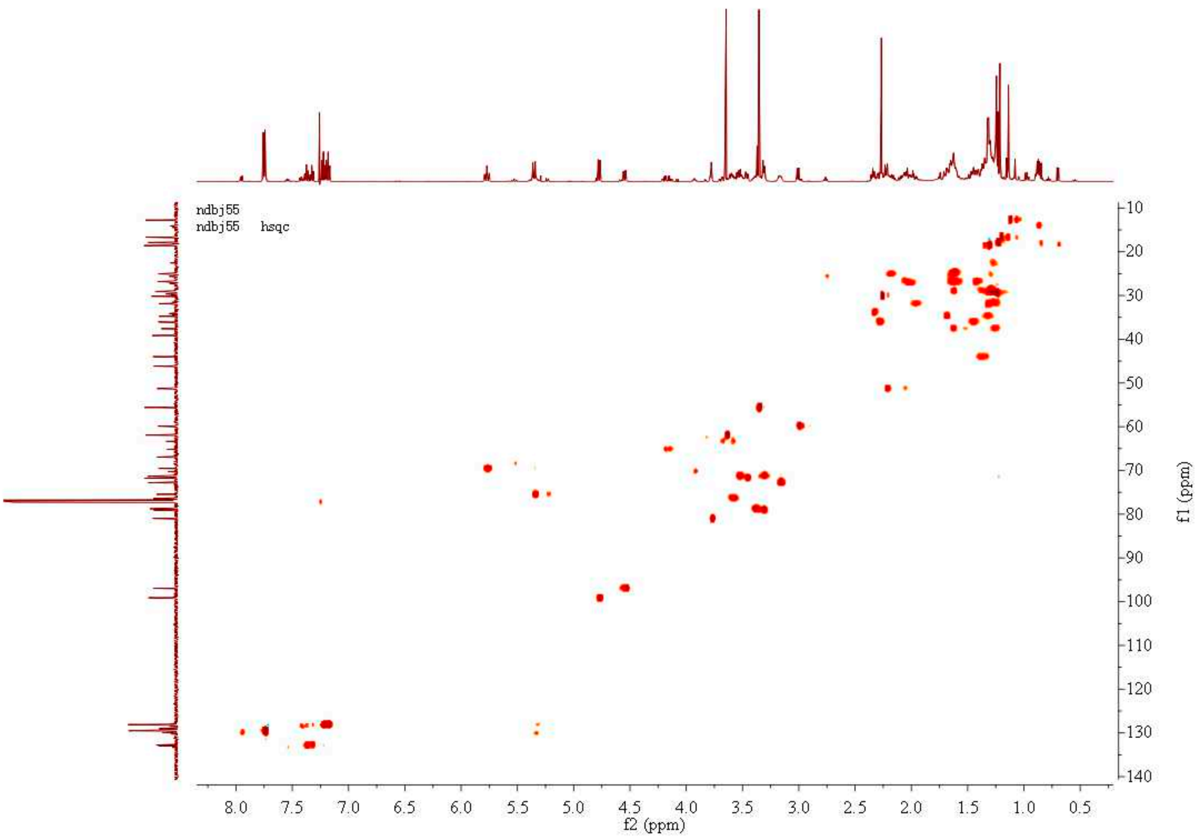

Figure S12 HSQC spectrum of compound **2**

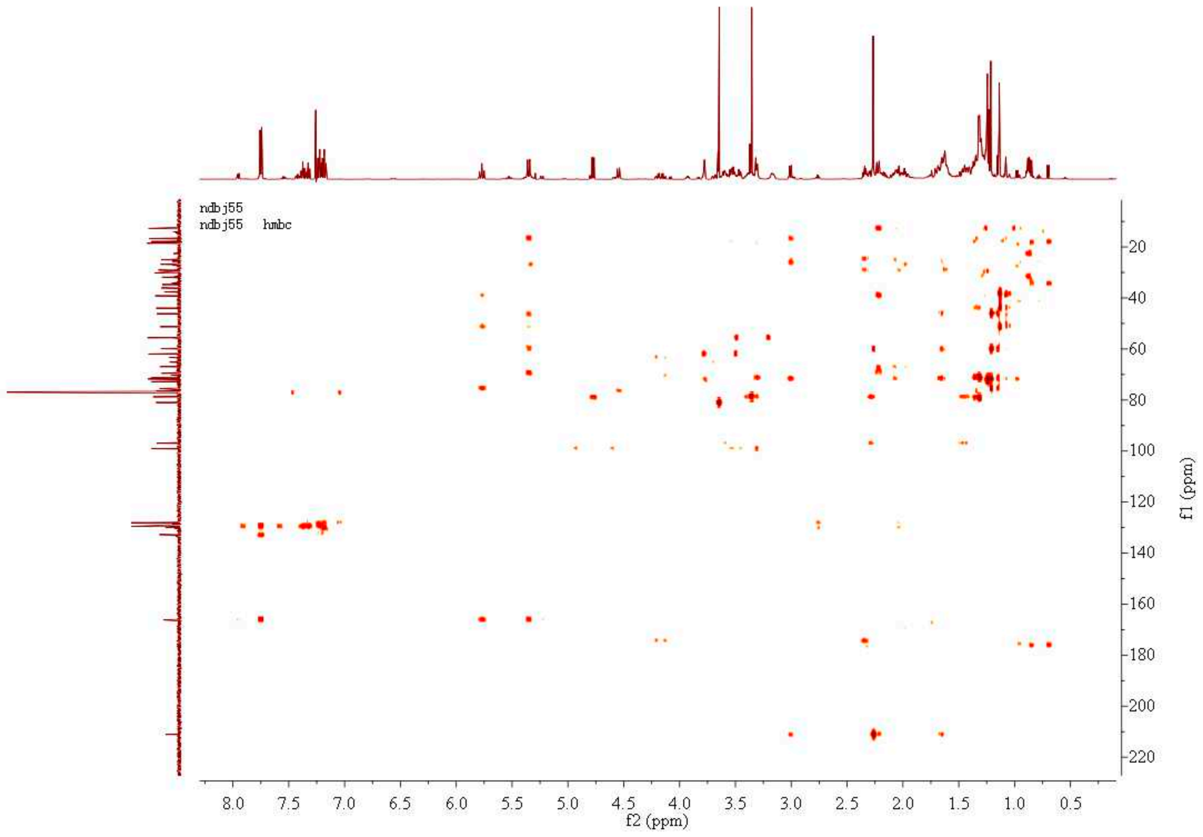

Figure S13 HMBC spectrum of compound **2**

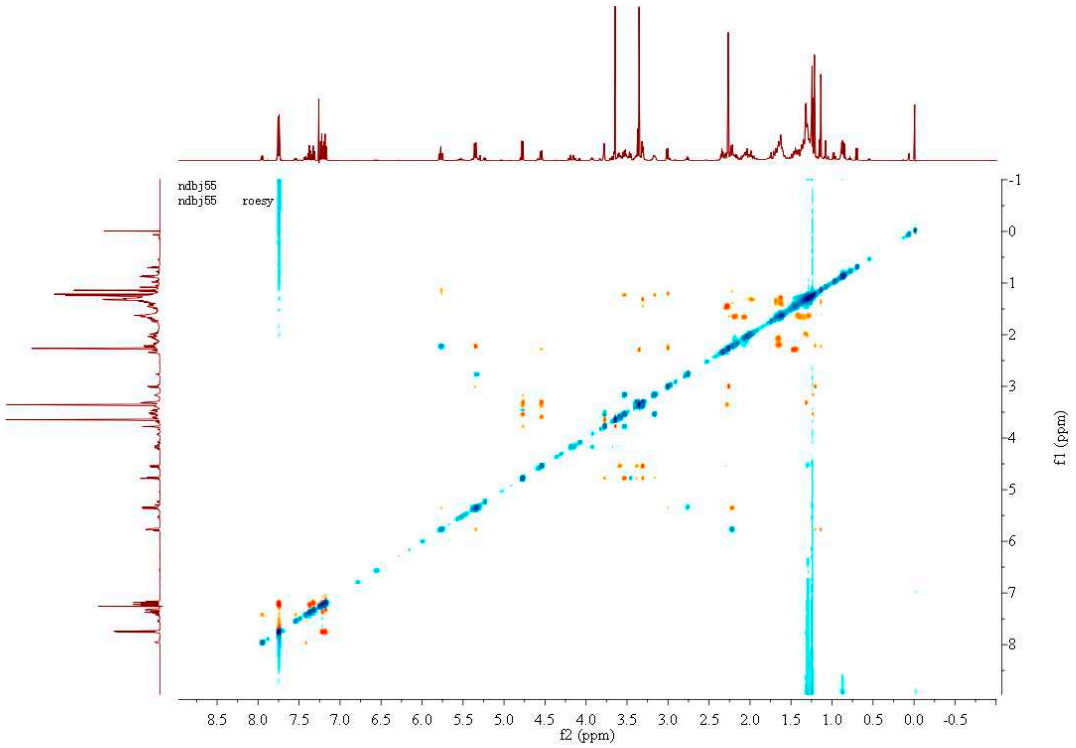

Figure S14 ROESY spectrum of compound **2**

Qualitative Analysis Report

**Data Filename**	ndbj-18.d	**Sample Name**	ndbj-18
**Sample Type**	Sample	**Position**	P1-B1
**Instrument Name**	Instrument 1	**User Name**	
**Acq Method**	s.m	**Acquired Time**	12/25/2020 3:23:45 PM
**IRM Calibration Status**	Success	**DA Method**	Default.m
**Comment**			

Sample Group **Info.**

Acquisition SW 6200 series TOF/6500 series
Version Q-TOF B.05.01 (B5125.2)

User Spectra

Fragmentor Voltage	Collision Energy	Ionization Mode
135	0	ESI

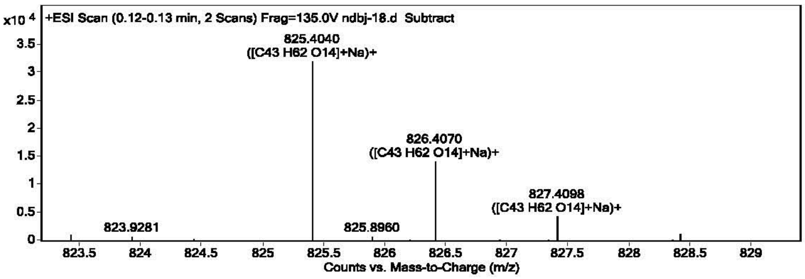

Peak List

m/z	z	Abund	Formula	Ion
347.2216	1	6409.36		
365.2326	1	7015		
499.2692	1	48087.03		
500.2729	1	15768.36		
691.3668	1	15865.02		
692.3689	1	6742.21		
820.4472	1	8841.49		
825.404	1	32109.97	C43 H62 O14	(M+Na)+
826.407	1	14198.66	C43 H62 O14	(M+Na)+
1359.7437	1	5780.44		

Formula Calculator Element Limits

Element	Min	Max
C	3	120
H	0	240
O	0	30

Formula Calculator Results

Formula	CalculatedMass	CalculatedMz	Mz	Diff. (mDa)	Diff. (ppm)	DBE
C43 H62 O14	802.4140	825.4032	825.4040	-0.80	-0.97	13.0000

--- End Of Report ---

Figure S15 HRESIMS spectrum of compound **3**

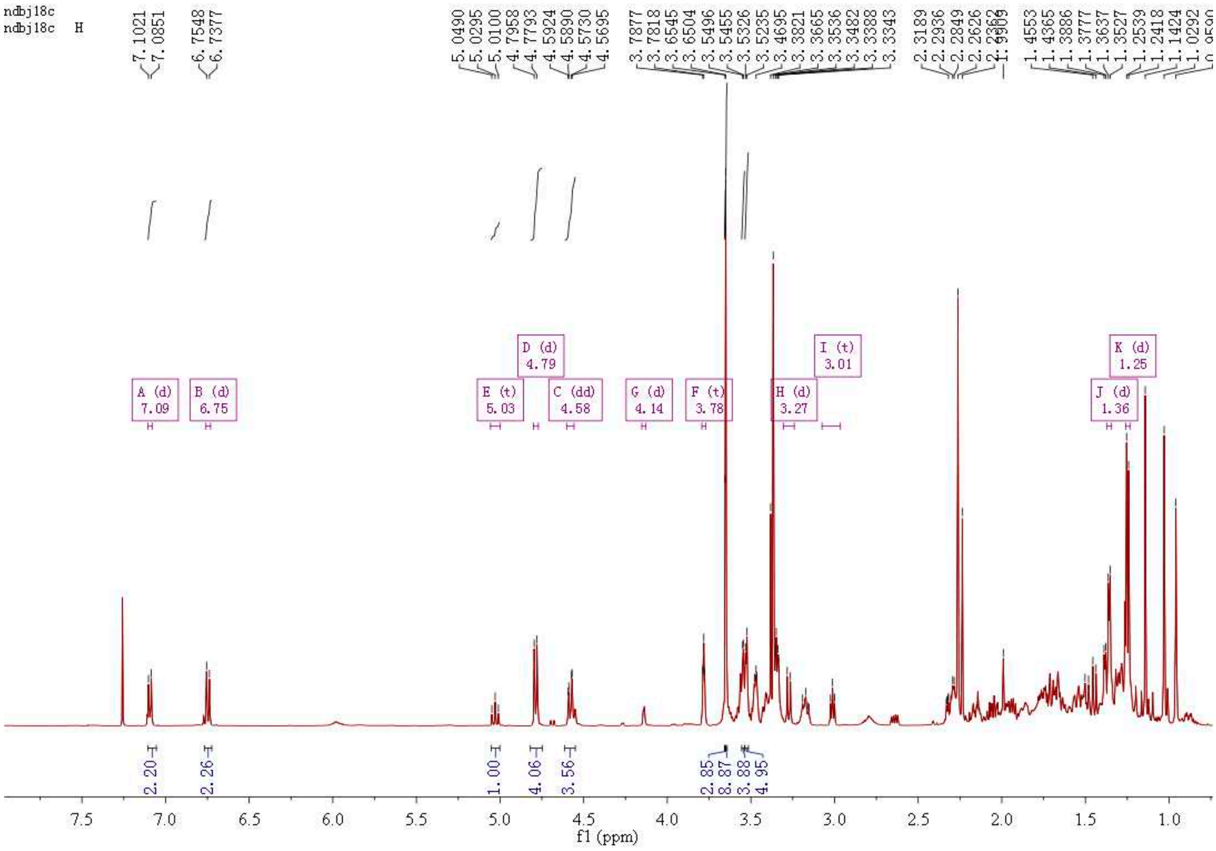

Figure S16 ^{1}H-NMR (500 MHz, $CDCl_3$) spectrum of compound **3**

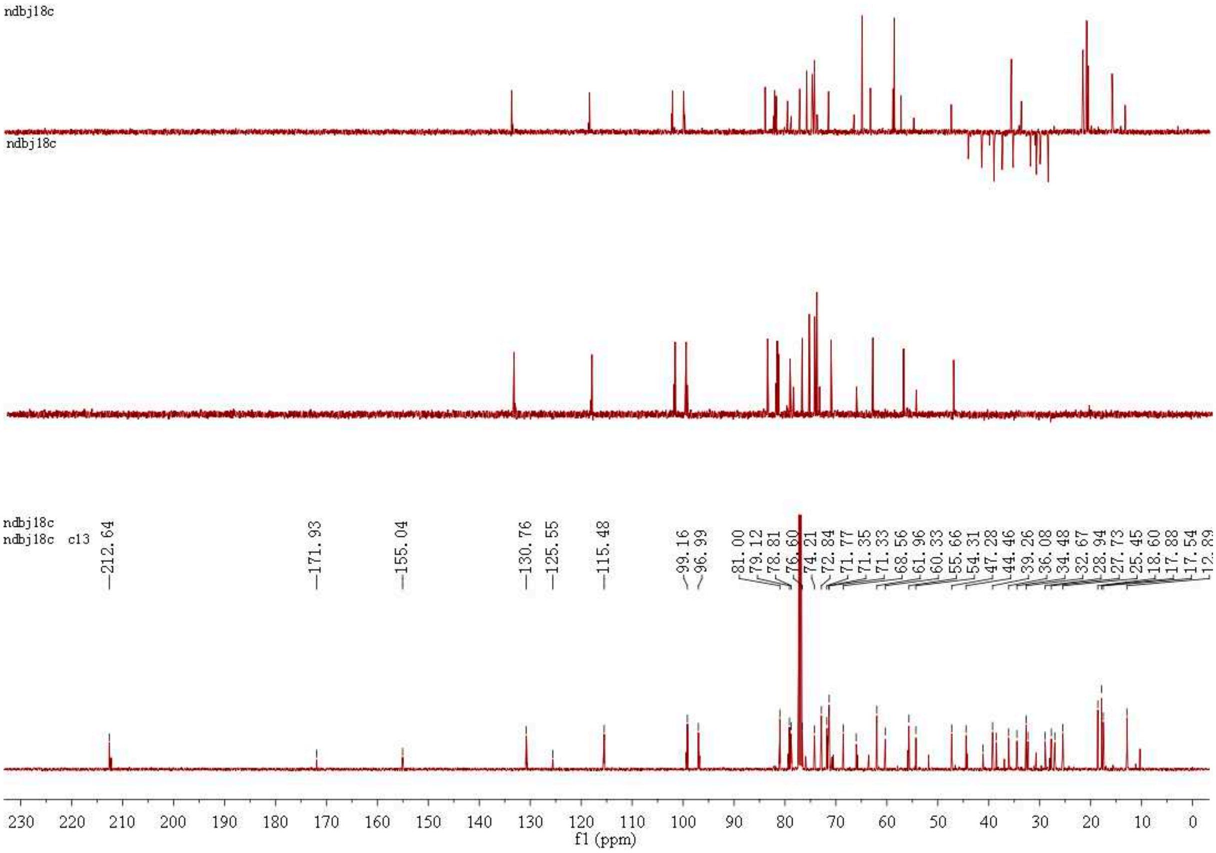

Figure S17 ^{13}C-NMR (125 MHz, $CDCl_3$) and DEPT spectrum of compound **3**

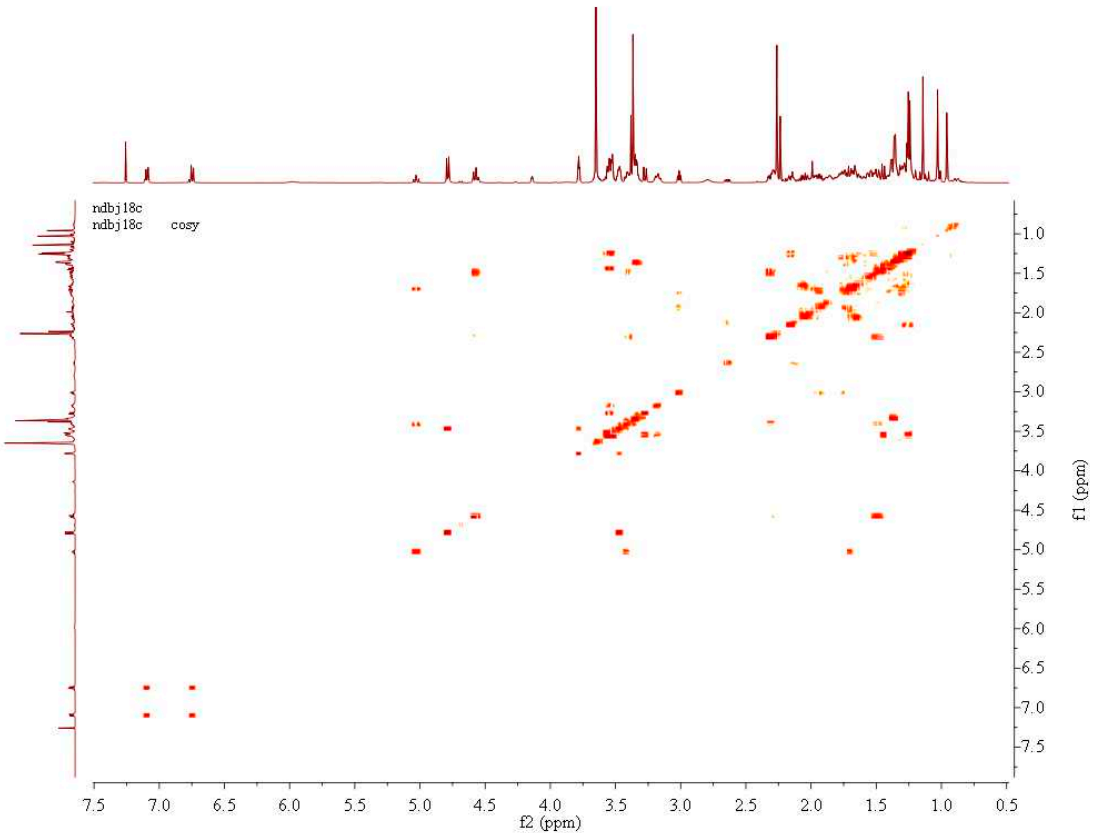

Figure S18 ^{1}H-^{1}H COSY spectrum of compound **3**

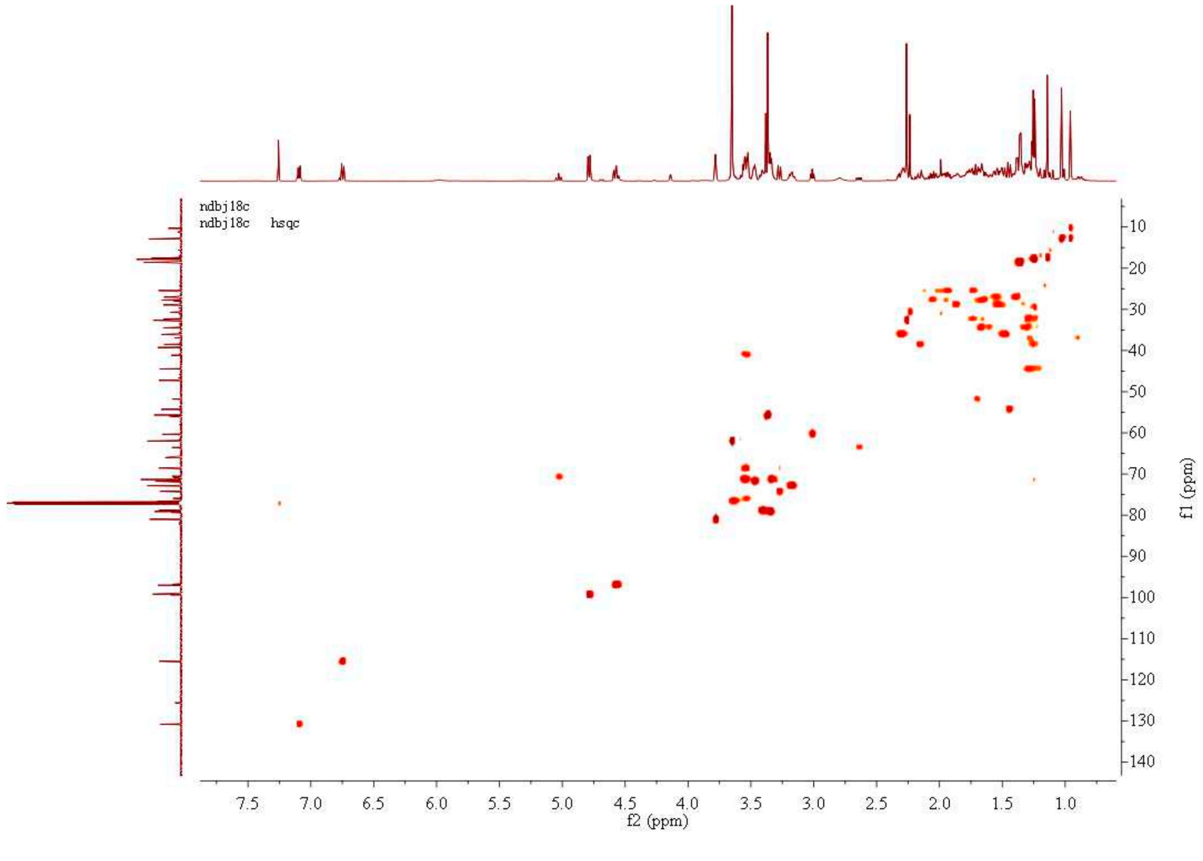

Figure S19 HSQC spectrum of compound **3**

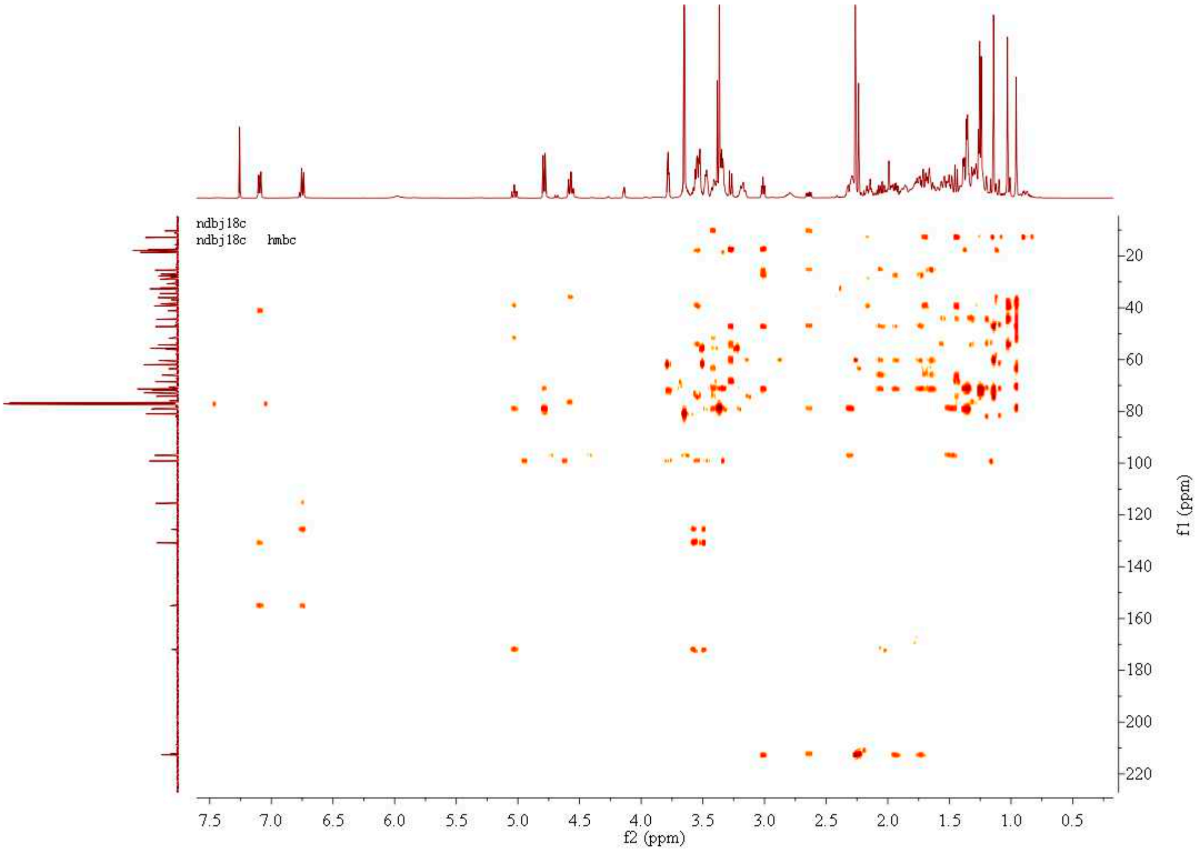

Figure S20 HMBC Spectrum of Compound **3**

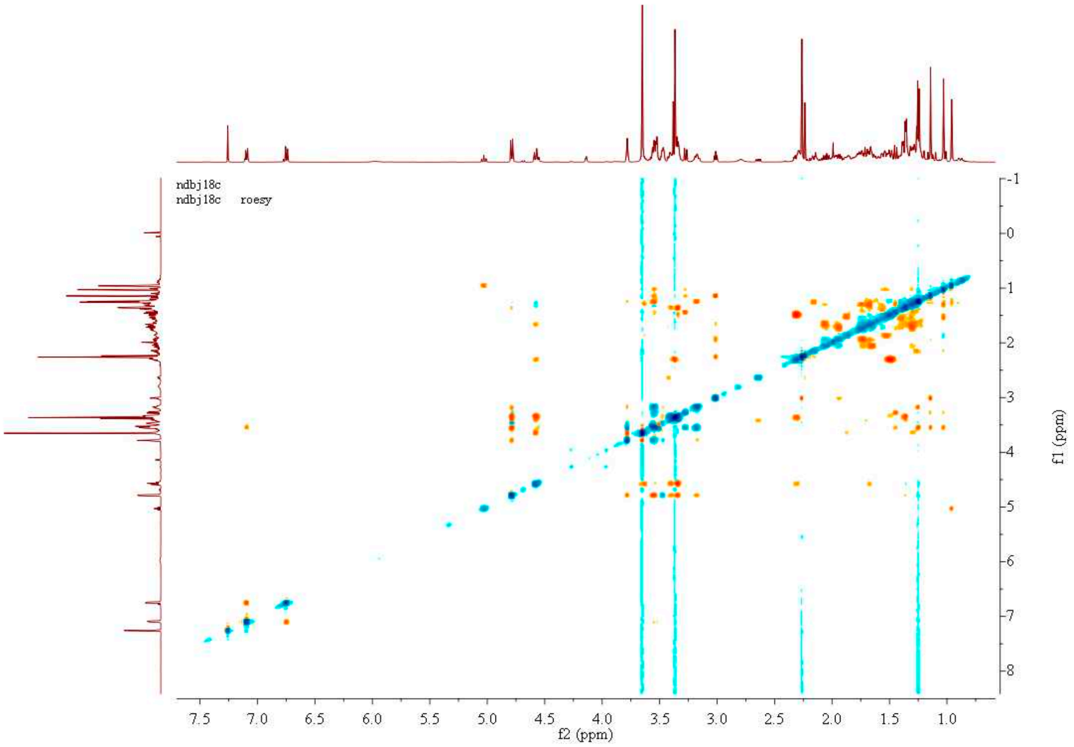

Figure S21 ROESY spectrum of compound **3**

Qualitative Analysis Report

**Data Filename**	ndbj-24.d	**Sample Name**	ndbj-24
**Sample Type**	Sample	**Position**	P1-A2
**Instrument Name**	Instrument 1	**User Name**	
**Acq Method**	s.m	**Acquired Time**	11/26/2020 3:37:32 PM
**IRM Calibration Status**	Success	**DA Method**	Default.m
**Comment**			

**Sample Group**		**Info.**
**Acquisition SW Version**	6200 series TOF/6500 series Q-TOF B.05.01 (B5125.2)	

User Spectra

Fragmentor Voltage	Collision Energy	Ionization Mode
175	0	ESI

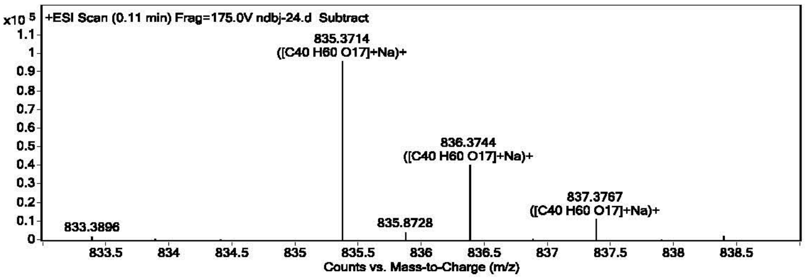

Peak List

m/z	z	Abund	Formula	Ion
830.4153	1	48589.8		
831.4181	1	21074.41		
835.3714	1	96274.69	C40 H60 O17	(M+Na)+
836.3744	1	40454.15	C40 H60 O17	(M+Na)+
953.4694	1	16813.27		
955.4812	1	11500.18		
995.4805	1	33026.6		
996.4838	1	16801.98		
1647.7493	1	24481.94		
1648.7539	1	21411.65		

Formula Calculator Element Limits

Element	Min	Max
C	3	60
H	0	120
O	0	30

Formula Calculator Results

Formula	CalculatedMass	CalculatedMz	Mz	Diff. (mDa)	Diff. (ppm)	DBE
C40 H60 O17	812.3831	835.3723	835.3714	0.90	1.08	11.0000

--- End Of Report ---

Figure S22 HRESIMS spectrum of compound **4**

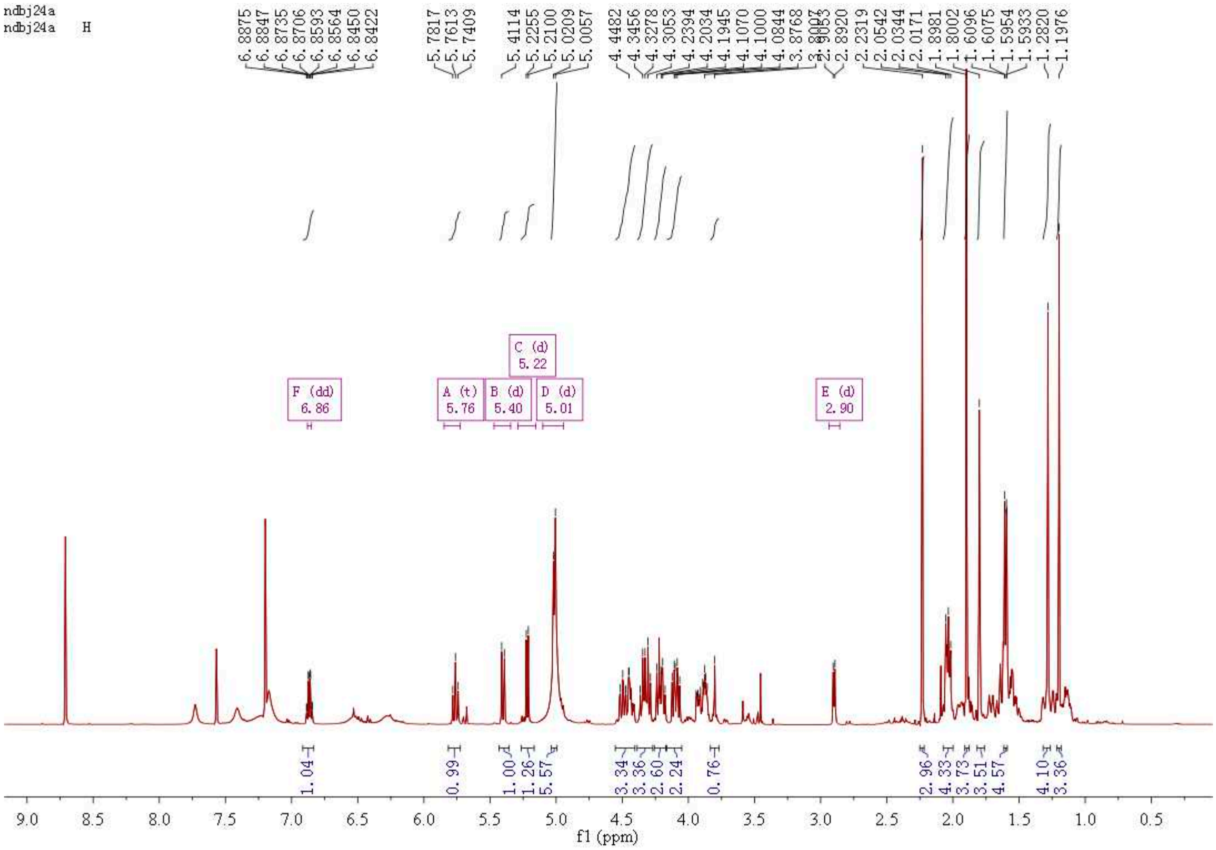

Figure S23 ^{1}H-NMR (500 MHz, pyridine-d_5) spectrum of compound **4**

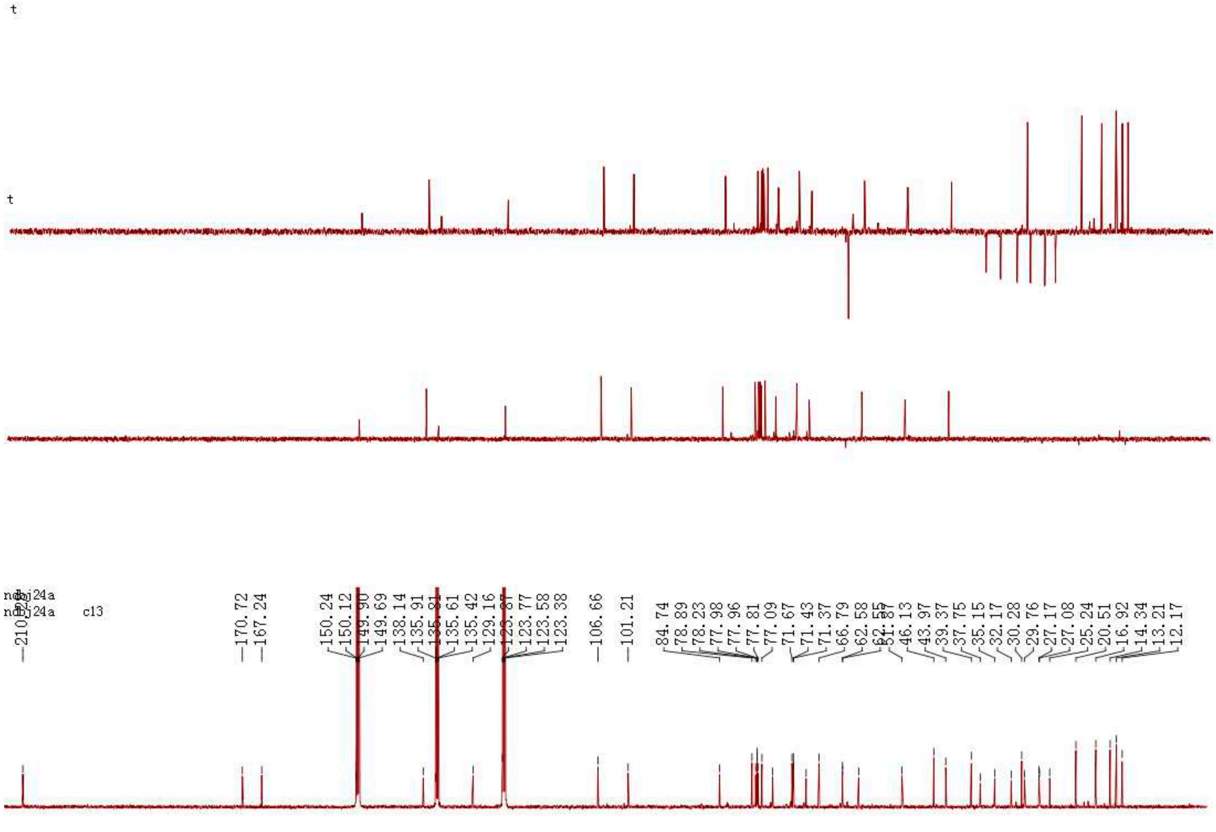

Figure S24 ^{13}C-NMR and DEPT (125 MHz, pyridine-d_5) spectrum of compound **4**

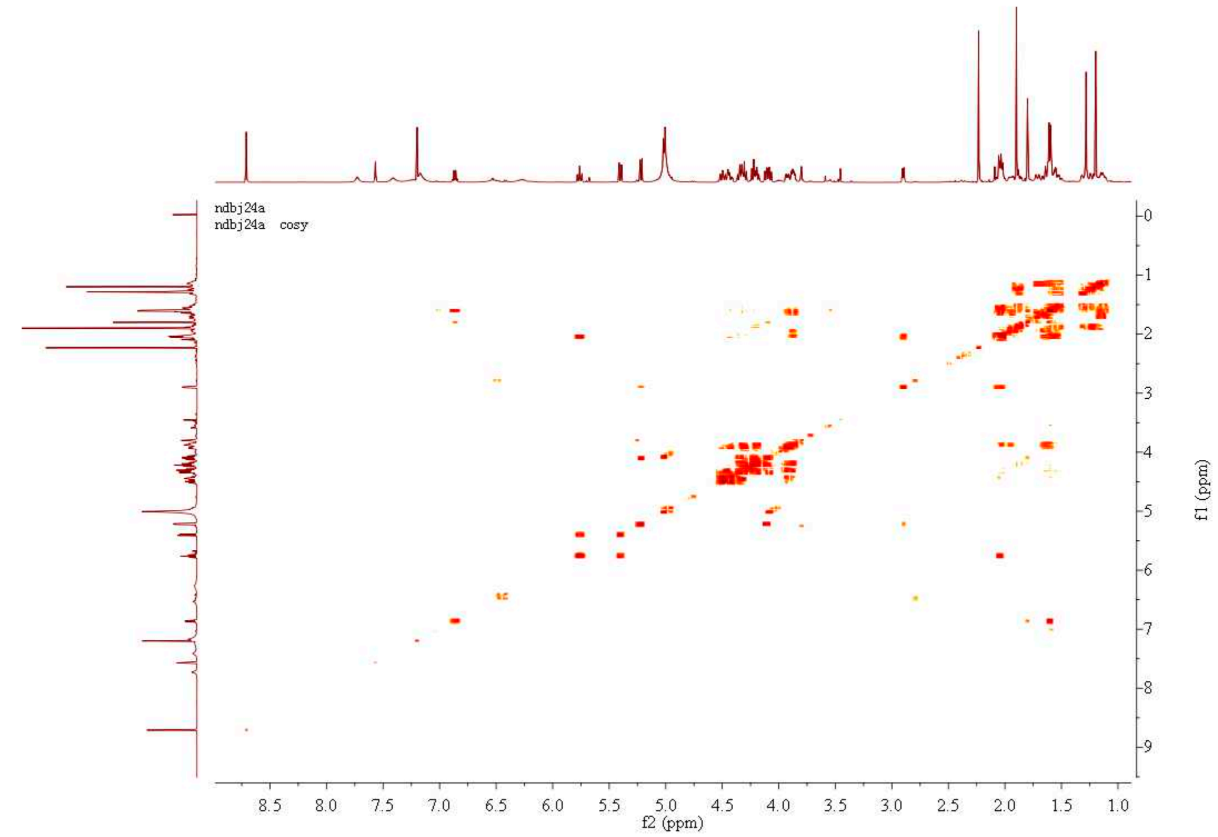

Figure S25 ^{1}H-^{1}H COSY spectrum of compound **4**

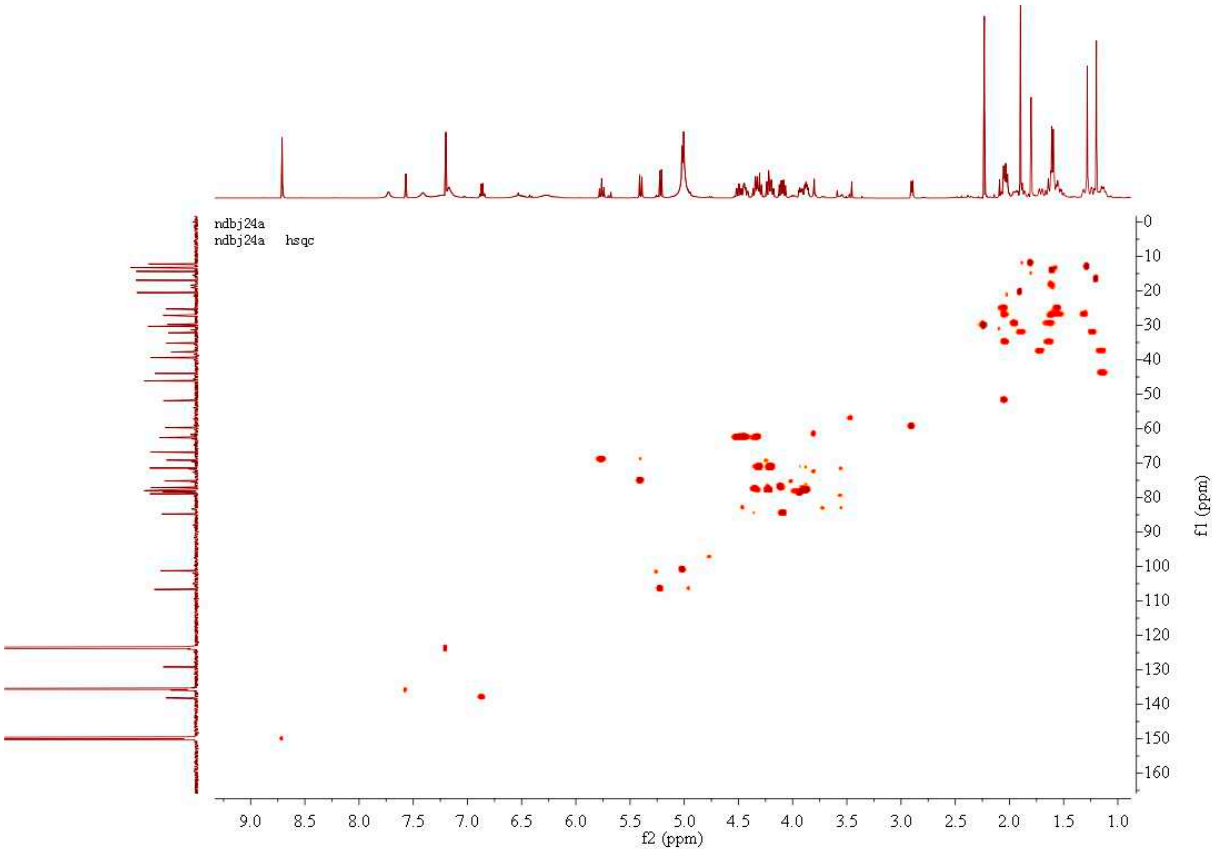

Figure S26 HSQC spectrum of compound **4**

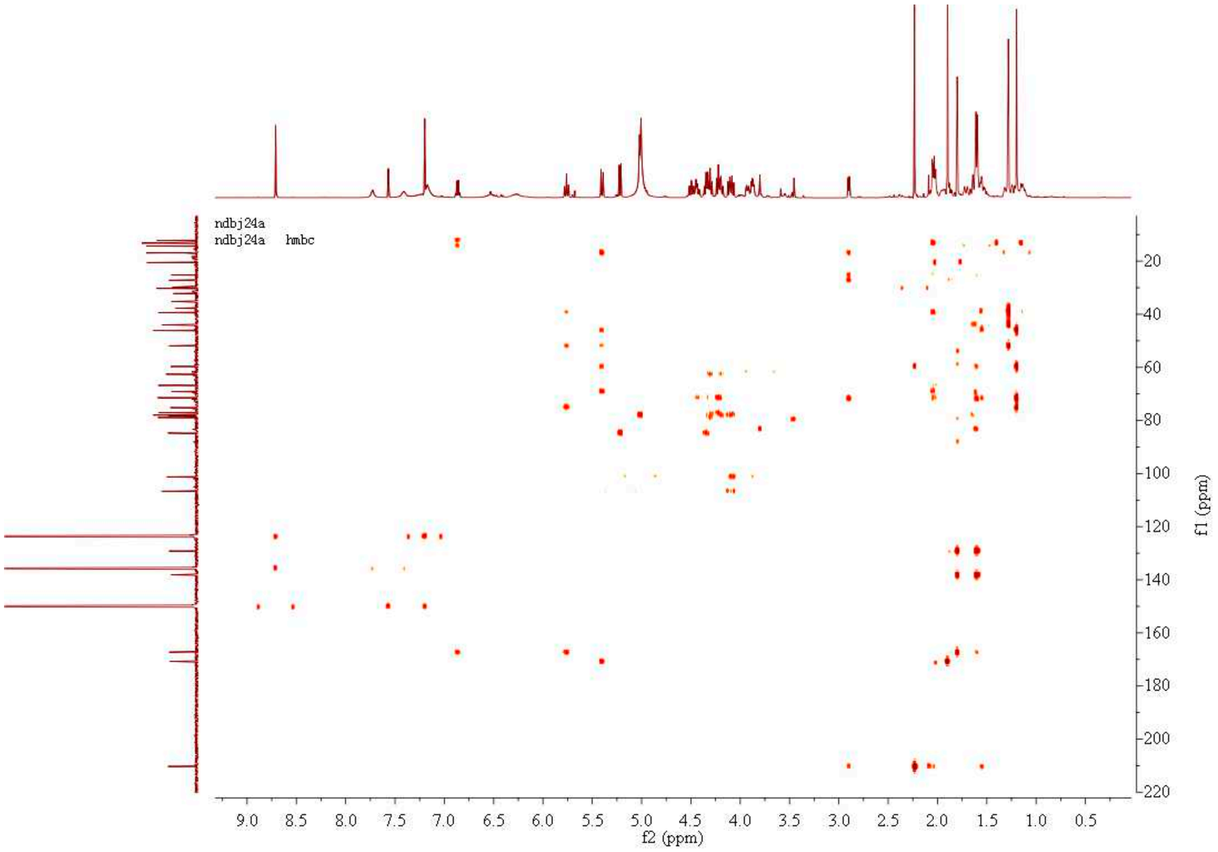

Figure S27 The HMBC spectrum of compound **4**

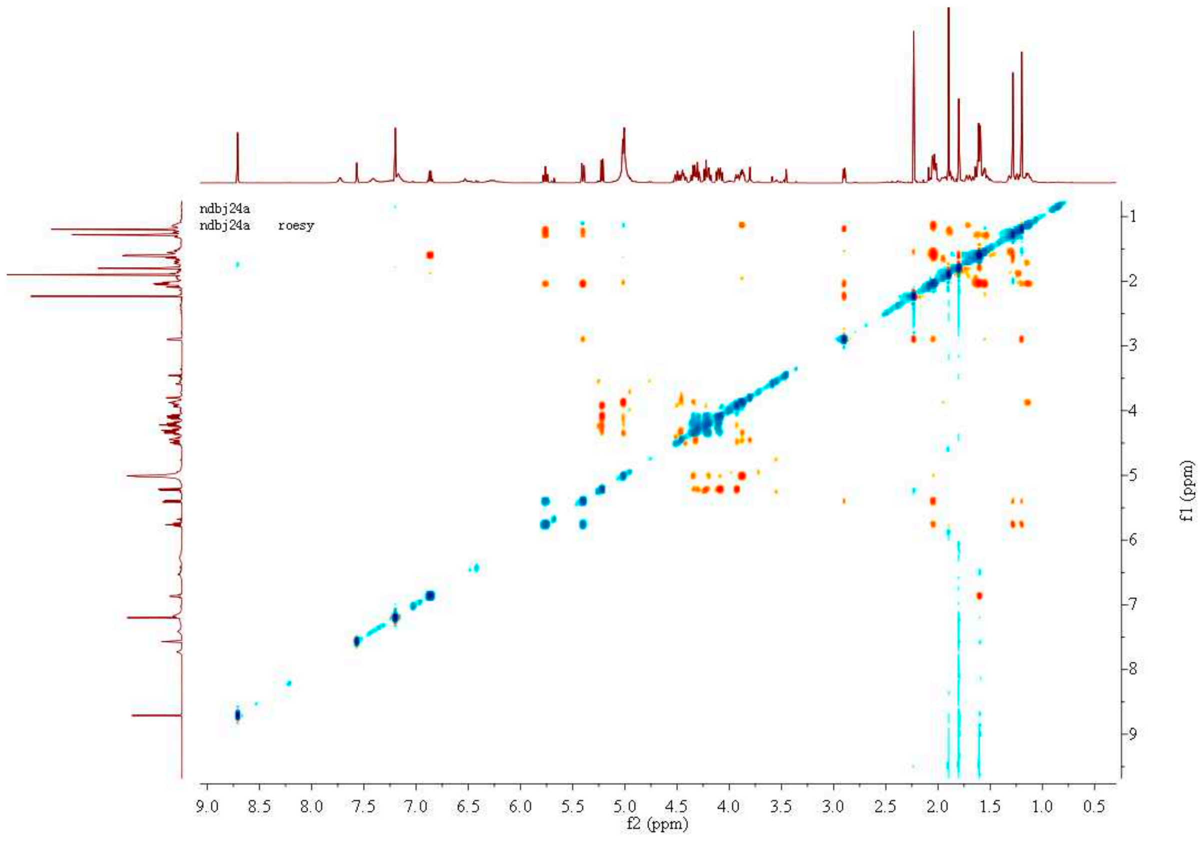

Figure S28 ROESY spectrum of compound **4**

Qualitative Analysis Report

**Data Filename**	ndbj-25.d	**Sample Name**	ndbj-25
**Sample Type**	Sample	**Position**	P1-C1
**Instrument Name**	Instrument 1	**User Name**	
**Acq Method**	s.m	**Acquired Time**	11/27/2020 3:26:00 PM
**IRM Calibration Status**	Success	**DA Method**	Default.m
**Comment**			

**Sample Group**		**Info.**
**Acquisition SW Version**	6200 series TOF/6500 series Q-TOF B.05.01 (B5125.2)	

User Spectra

Fragmentor Voltage	Collision Energy	Ionization Mode
135	0	ESI

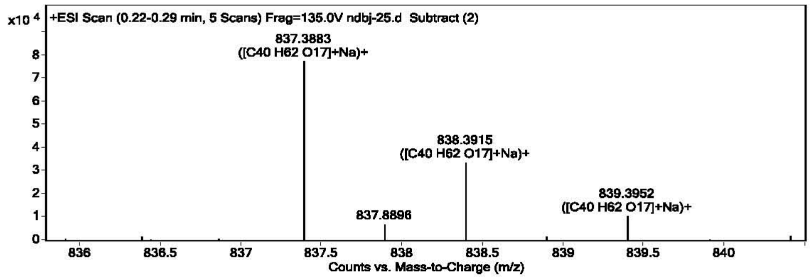

Peak List

m/z	z	Abund	Formula	Ion
112.1875		30322.51		
832.4326	1	52515.62		
837.3883	1	77573.45	C40 H62 O17	(M+Na)+
838.3915	1	33777	C40 H62 O17	(M+Na)+
853.4192	1	148654.5		
854.4221	1	65545.1		
955.4875	1	100281.19		
956.4909	1	50290.4		
1651.7858	1	27988.96		
1652.7894	1	24435.12		

Formula Calculator Element Limits

Element	Min	Max
C	3	60
H	0	120
O	0	30

Formula Calculator Results

Formula	CalculatedMass	CalculatedMz	Mz	Diff. (mDa)	Diff. (ppm)	DBE
C40 H62 O17	814.3987	837.3879	837.3883	-0.40	-0.48	10.0000

--- End Of Report ---

Figure S29 HRESIMS spectrum of compound **5**

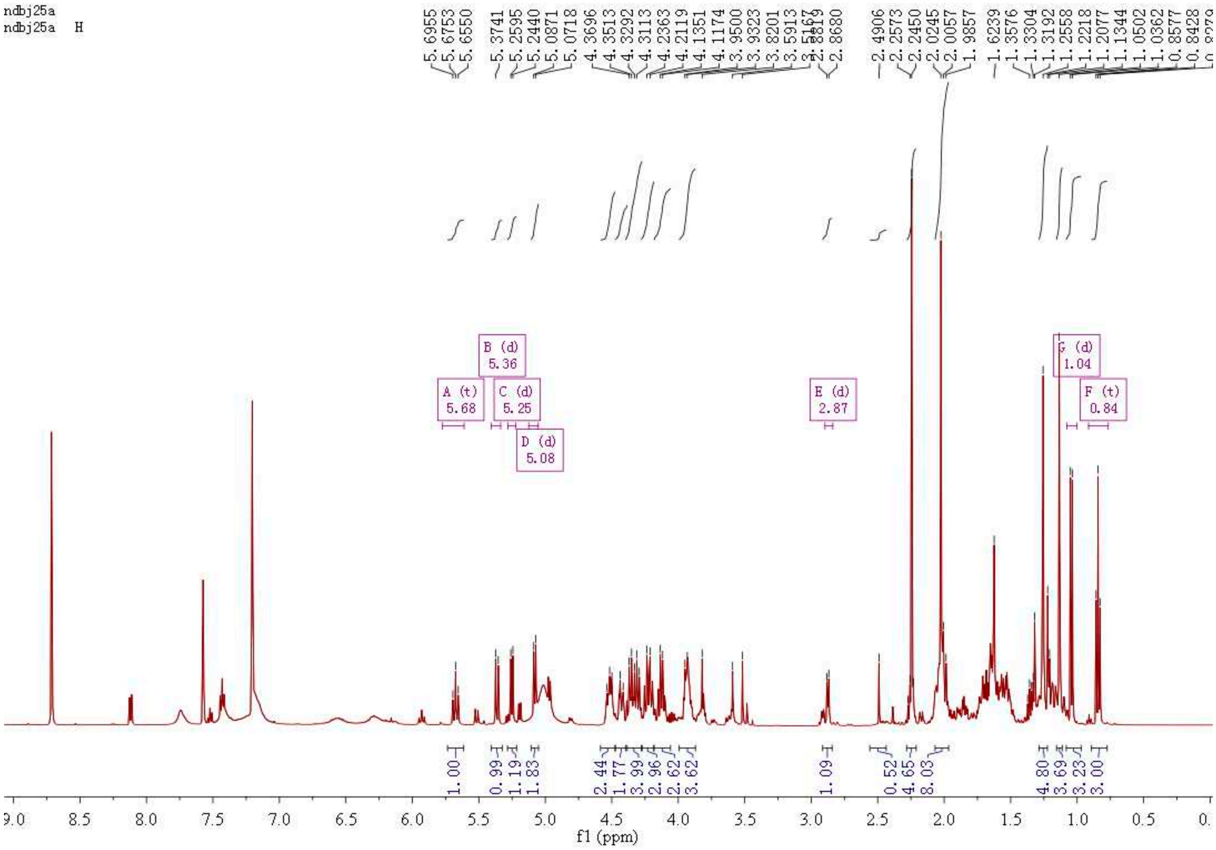

Figure S30 The ^{1}H-NMR (500 MHz, pyridine-d_5) spectrum of compound **5**

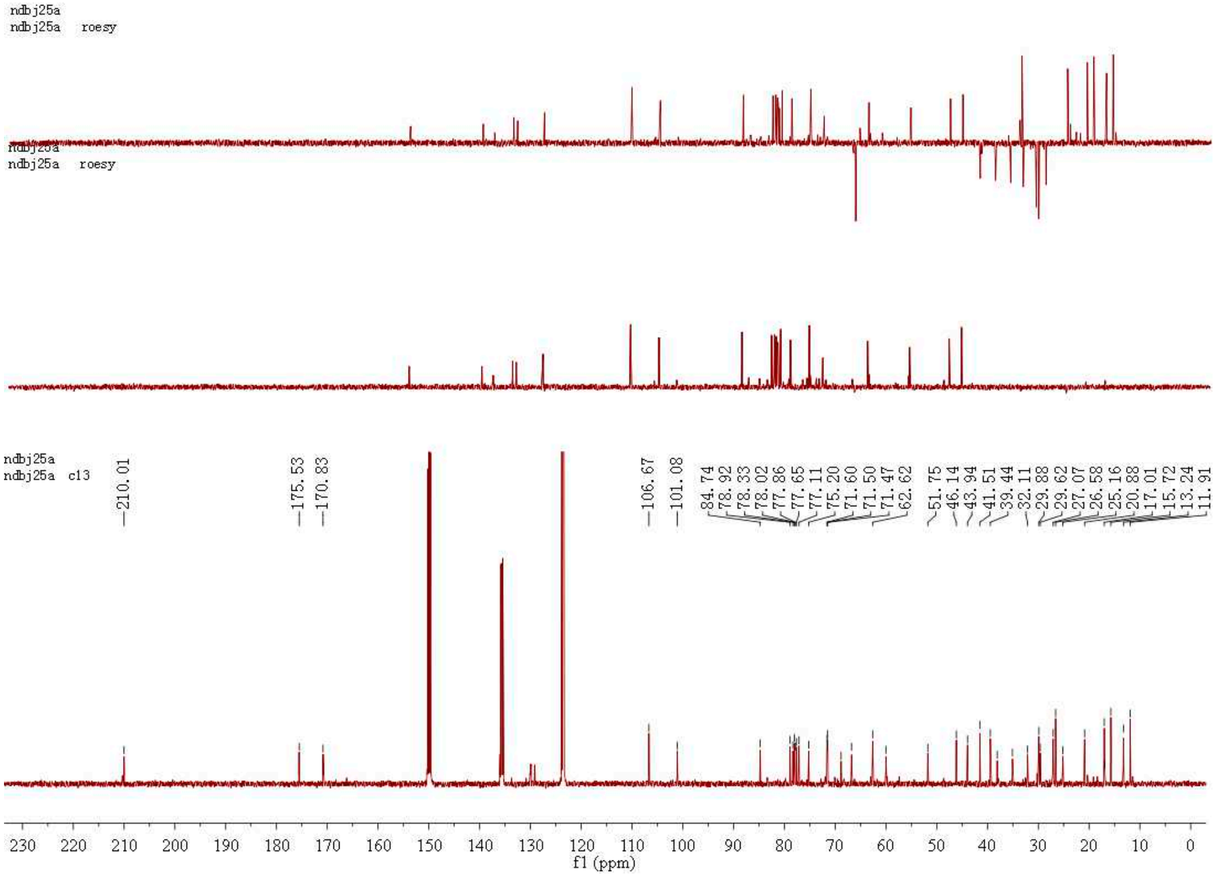

Figure S31 ^{13}C-NMR and DEPT (125 MHz, pyridine-d_5) spectrum of compound **5**

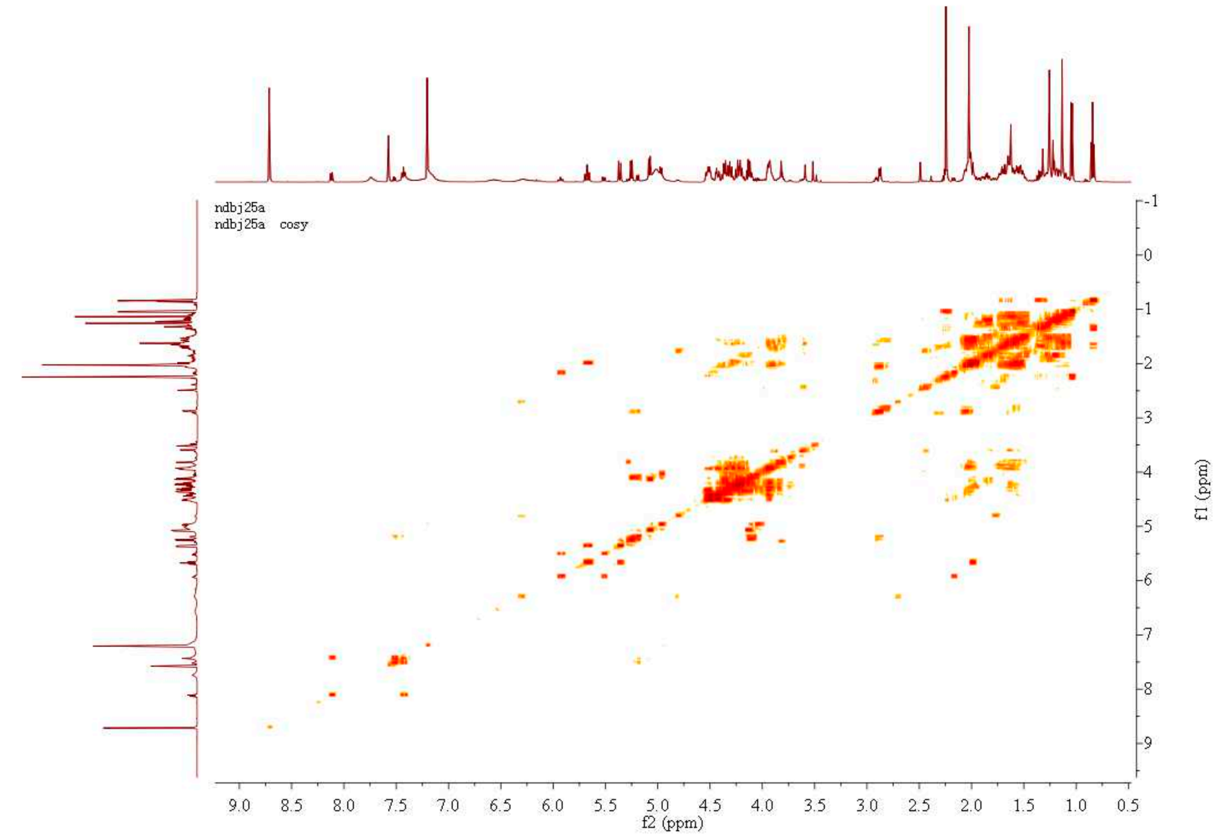

Figure S32 ^{1}H-^{1}H COSY spectrum of compound **5**

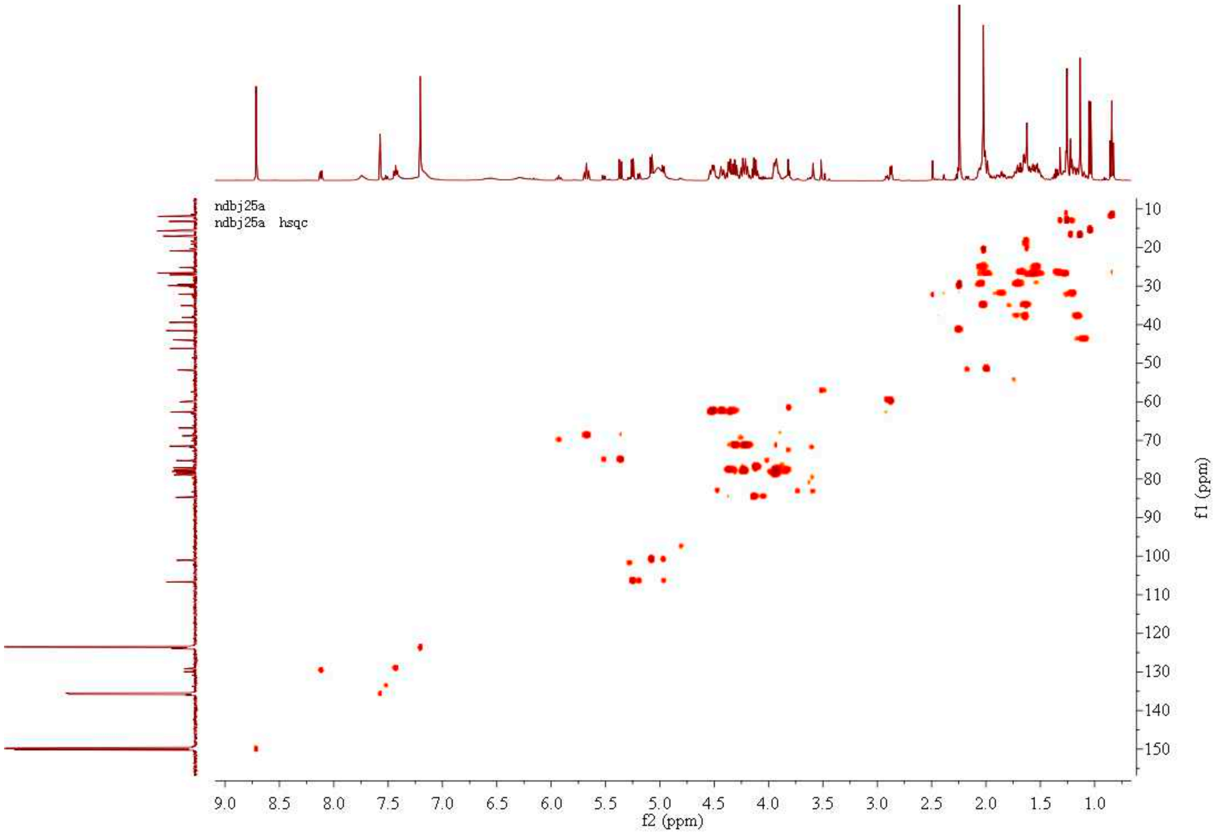

Figure S33 HSQC spectrum of compound **5**

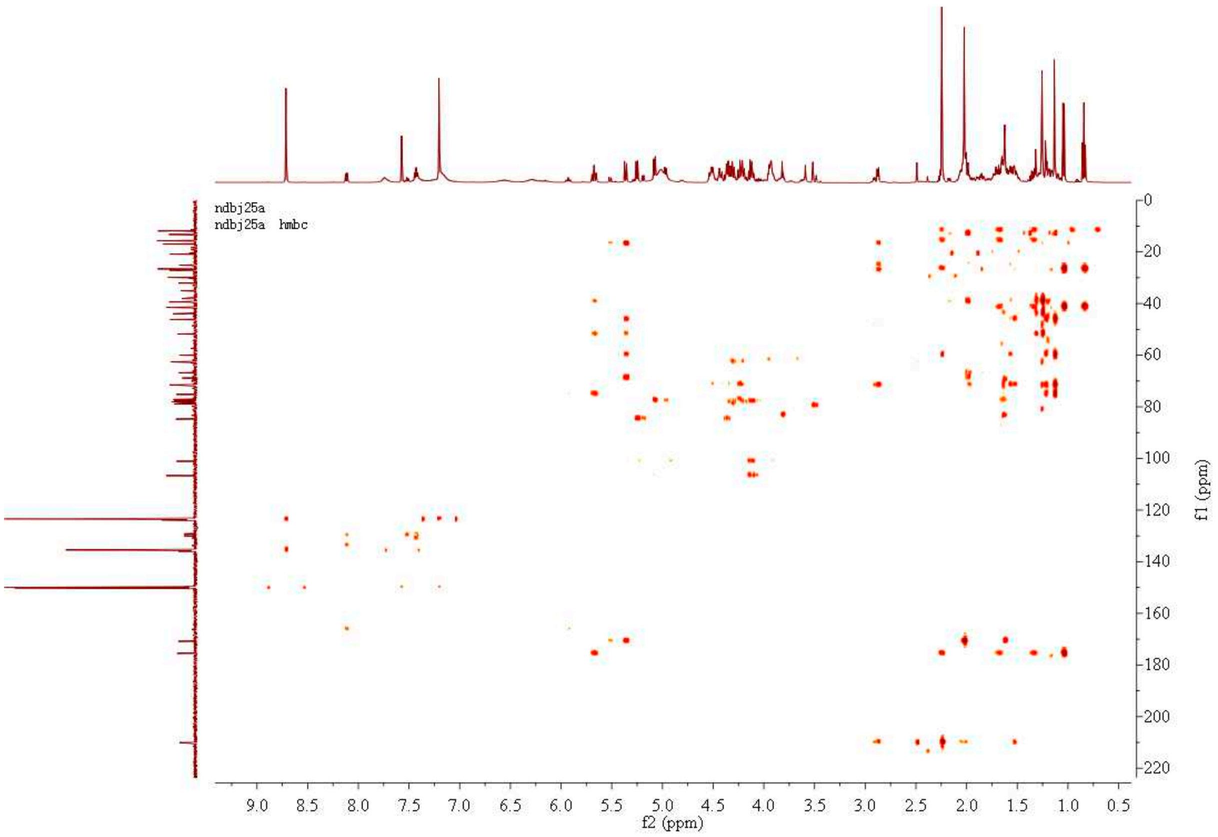

Figure S34 HMBC spectrum of compound **5**

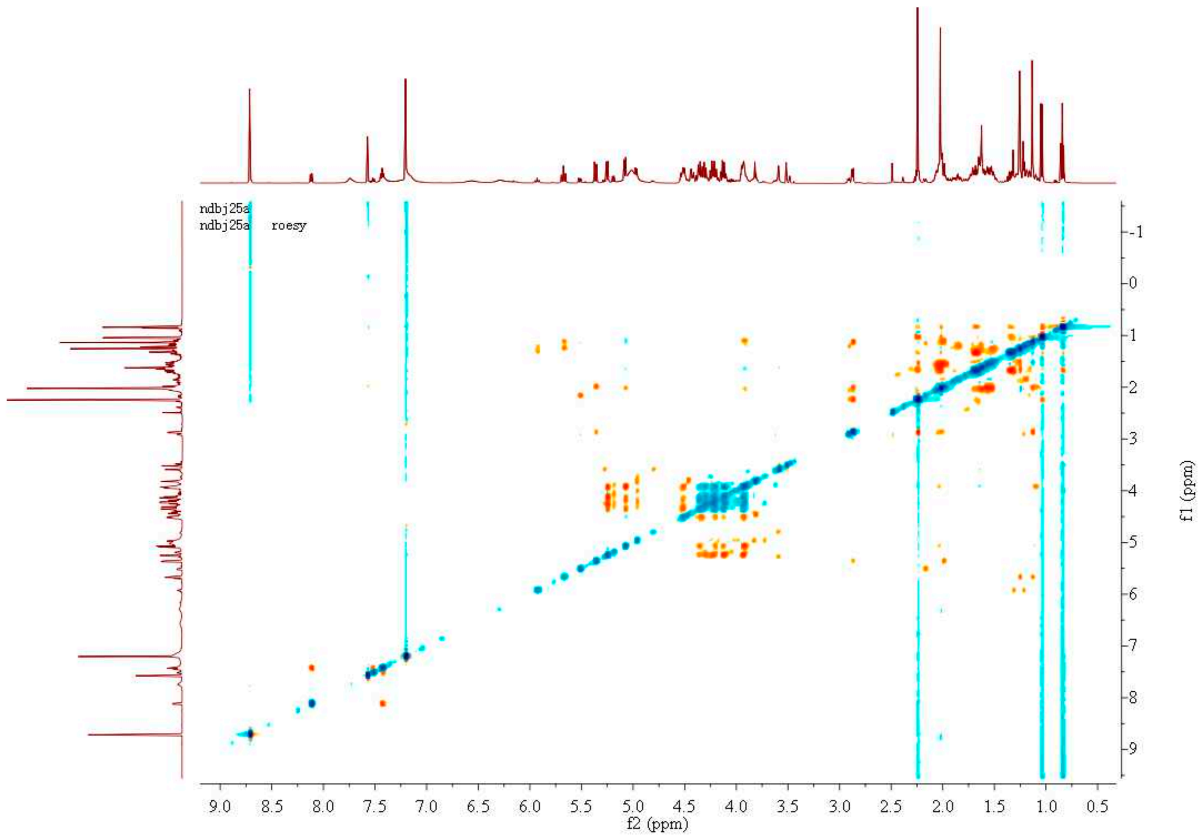

Figure S35 ROESY spectrum of compound **5**

Qualitative Analysis Report

**Data Filename**	ndbj-21.d	**Sample Name**	ndbj-21
**Sample Type**	Sample	**Position**	P1-A1
**Instrument Name**	Instrument 1	**User Name**	
**Acq Method**	s.m	**Acquired Time**	11/26/2020 3:36:21 PM
**IRM Calibration Status**	Success	**DA Method**	Default.m
**Comment**			

**Sample Group**		**Info.**
**Acquisition SW Version**	6200 series TOF/6500 series Q-TOF B.05.01 (B5125.2)	

User Spectra

Fragmentor Voltage	Collision Energy	Ionization Mode
175	0	ESI

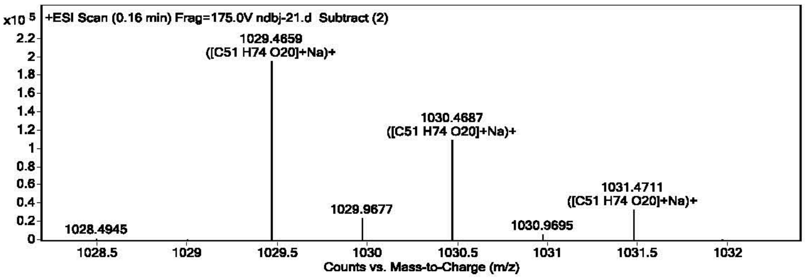

Peak List

m/z	z	Abund	Formula	Ion
541.2782	1	12301.04		
937.4756	1	40547.91		
938.4793	1	18861.75		
1024.5098	1	42988.28		
1025.5133	1	23825.68		
1029.4659	1	196133.84	C51 H74 O20	(M+Na)+
1029.9677	1	24163.45		
1030.4687	1	109558.27	C51 H74 O20	(M+Na)+
1031.4711	1	33537.78	C51 H74 O20	(M+Na)+
1045.4396	1	14389.06		

Formula Calculator Element Limits

Element	Min	Max
C	3	60
H	0	120
O	0	30

Formula Calculator Results

Formula	CalculatedMass	CalculatedMz	Mz	Diff. (mDa)	Diff. (ppm)	DBE
C51 H74 O20	1006.4773	1029.4666	1029.4659	0.70	0.68	15.0000

--- End Of Report ---

Figure S36 HRESIMS spectrum of compound **6**

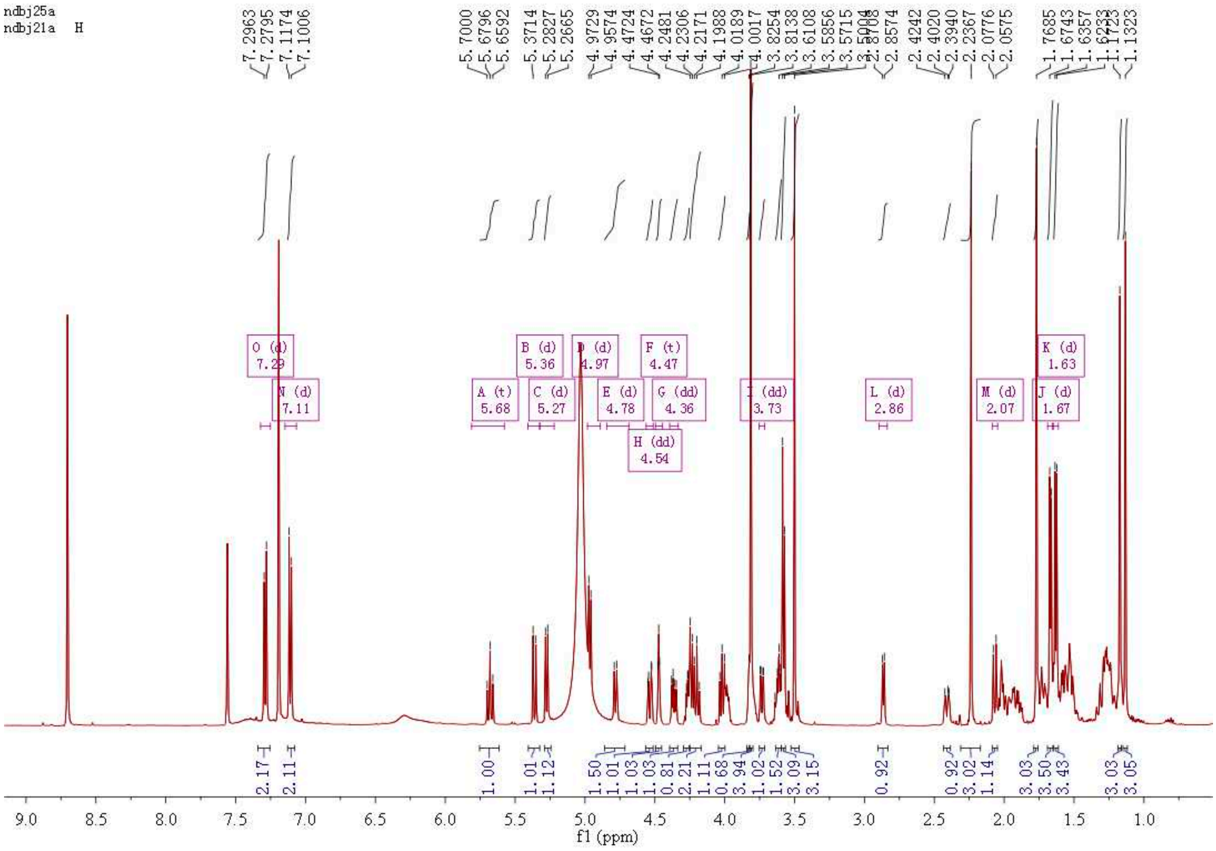

Figure S37 ^{1}H-NMR (500 MHz, pyridine-d_5) spectrum of compound **6**

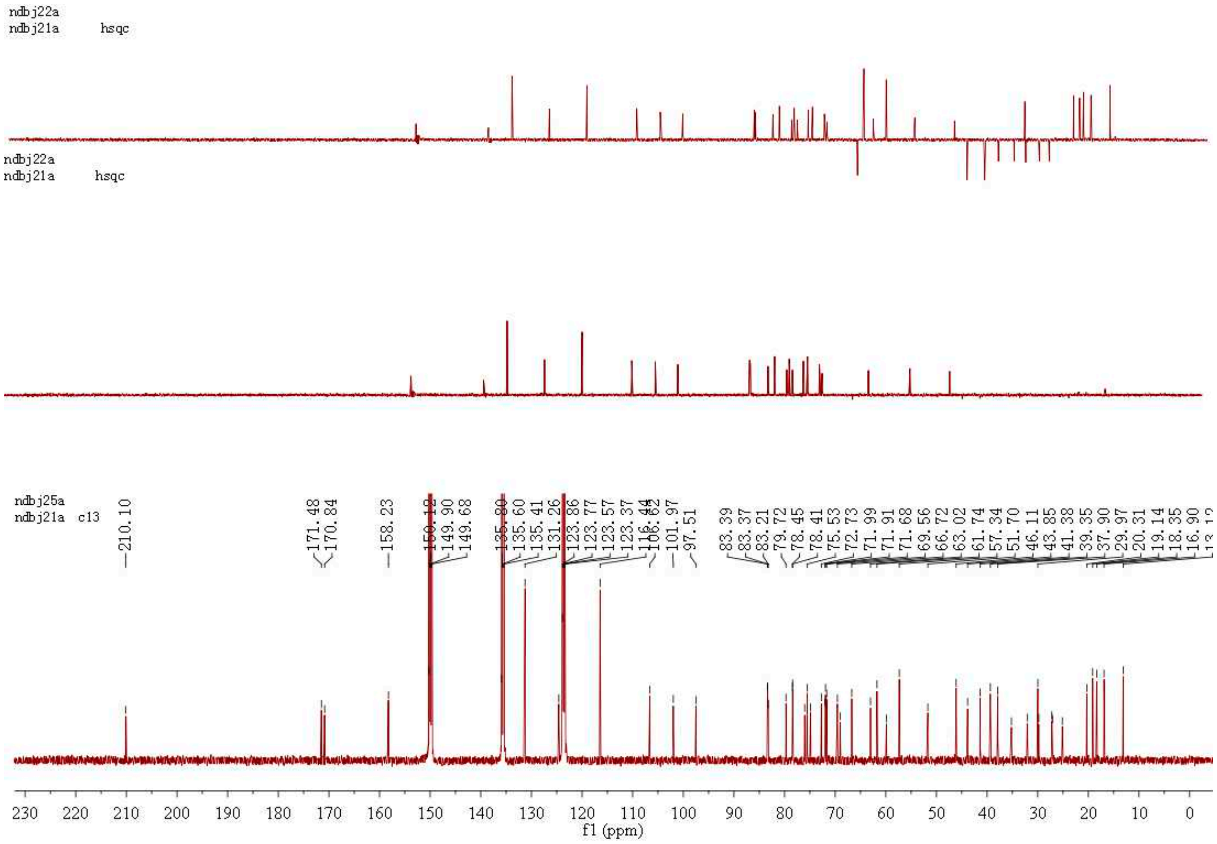

Figure S38 ^{13}C-NMR and DEPT (125 MHz, pyridine-d_5) spectrum of compound **6**

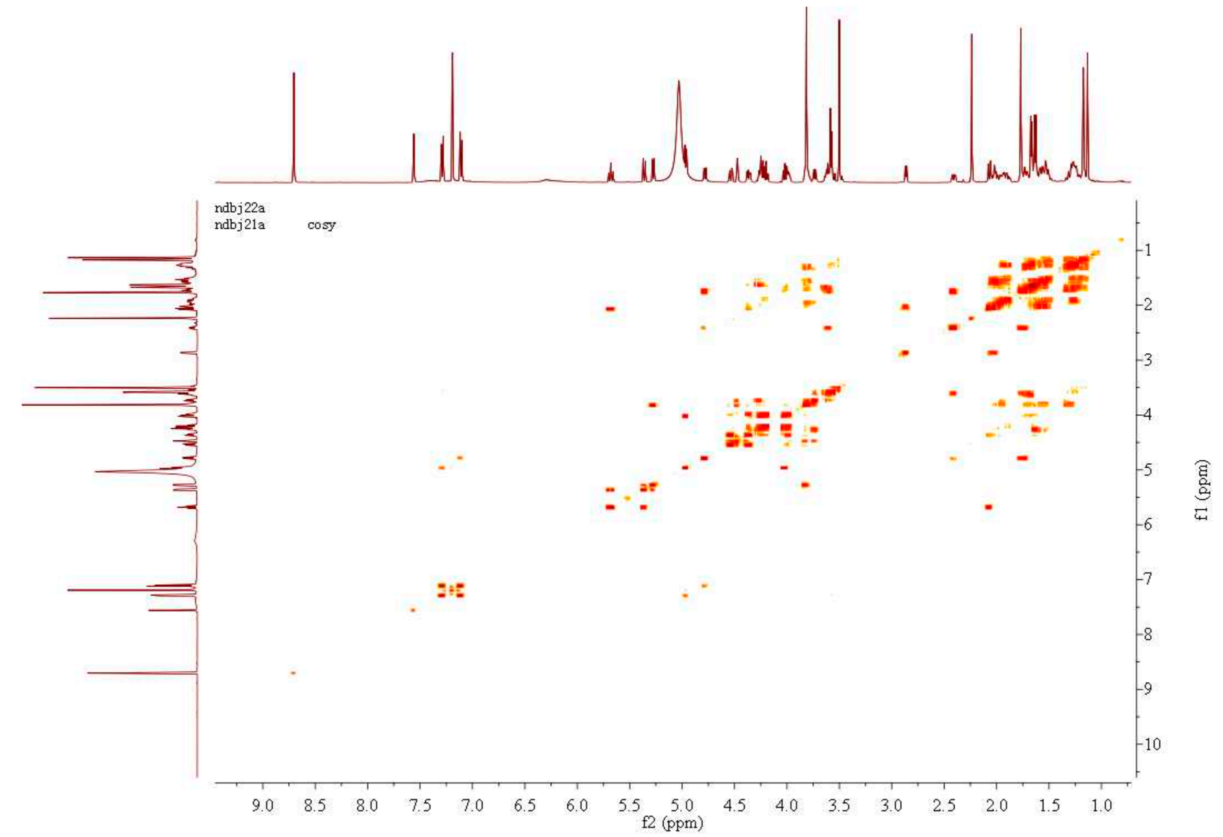

Figure S39 ^{1}H-^{1}H COSY spectrum of compound **6**

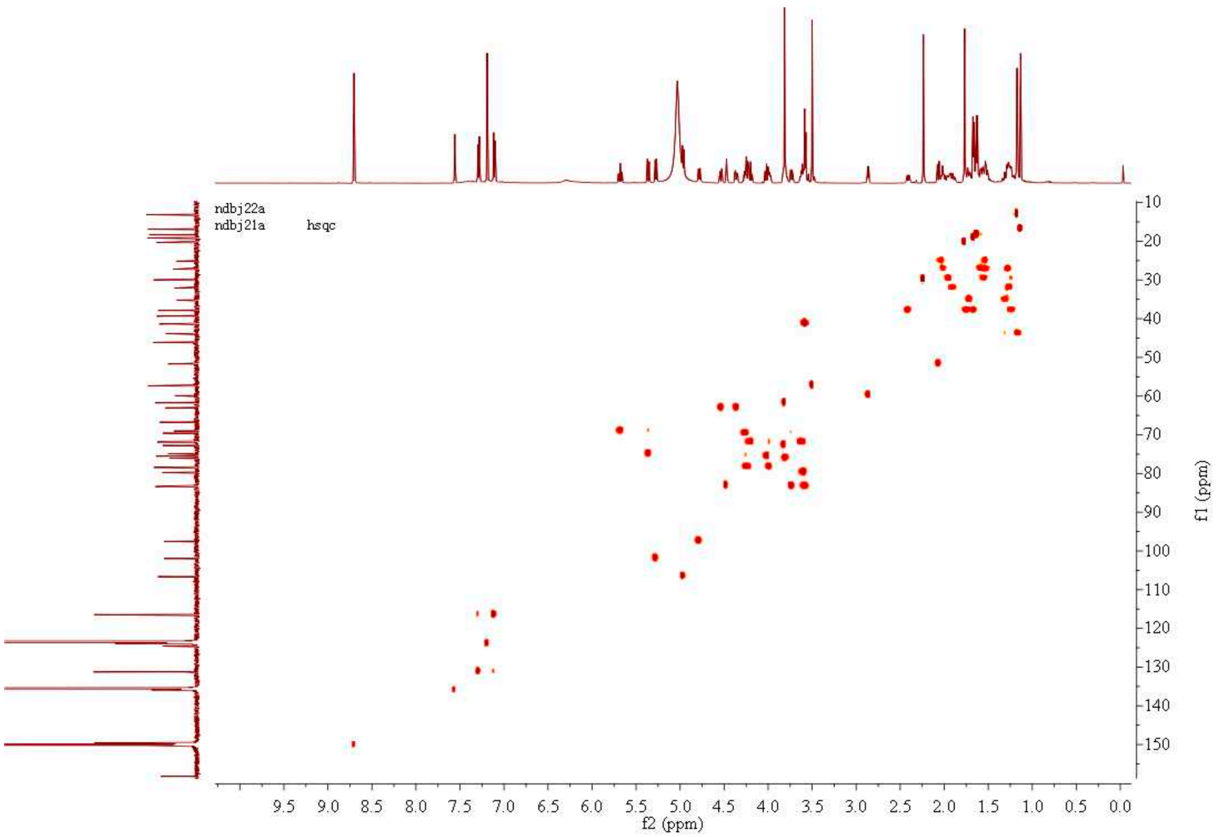

Figure S40 HSQC spectrum of compound **6**

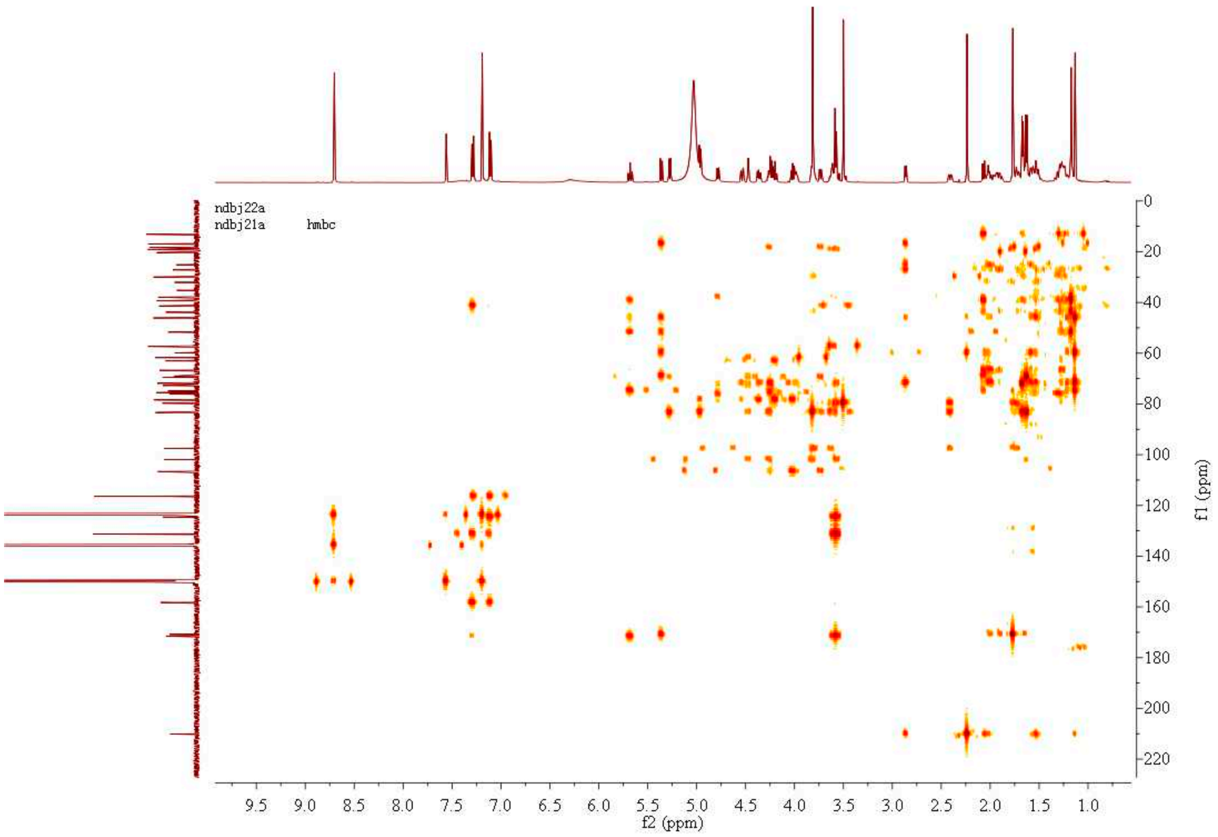

Figure S41 HMBC spectrum of compound **6**

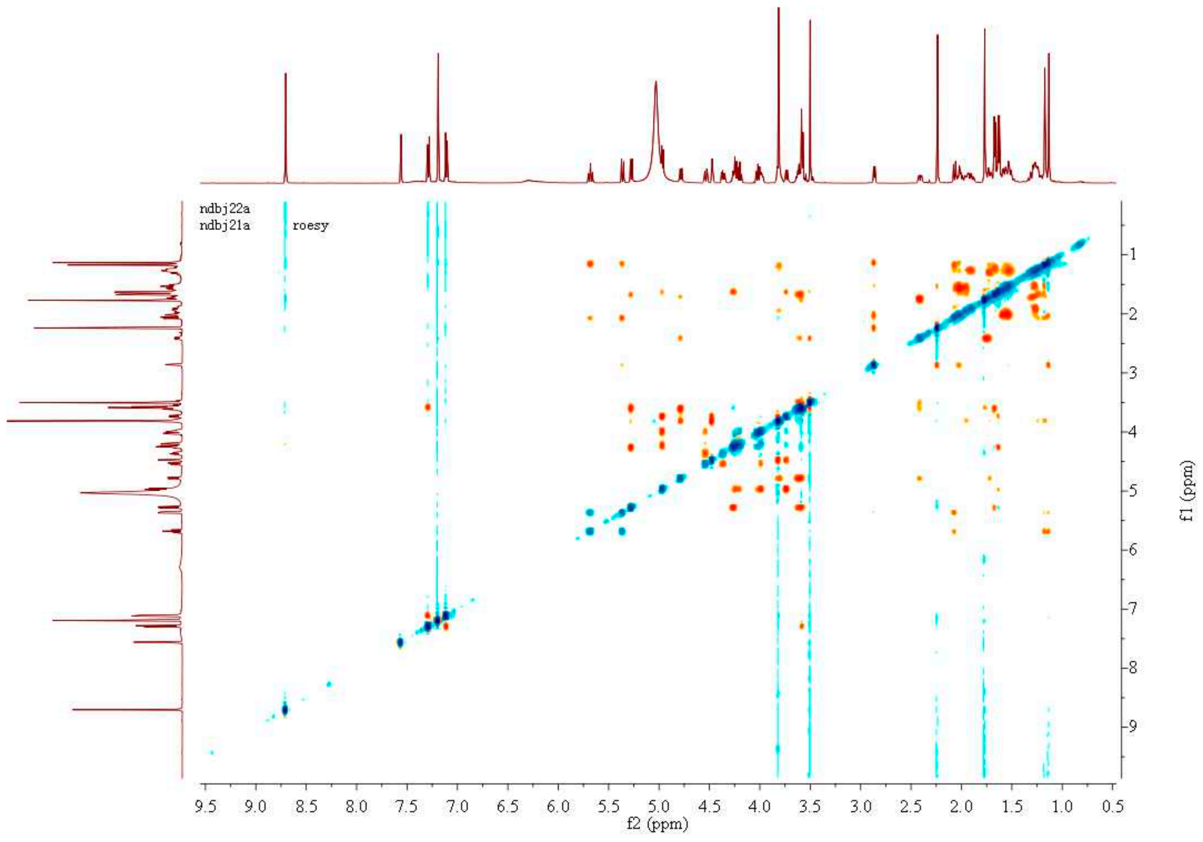

Figure S42 ROESY spectrum of compound **6**

Qualitative Analysis Report

**Data Filename**	20221124ESIA7.d	**Sample Name**	ndbj-43
**Sample Type**	Sample	**Position**	
**Instrument Name**	Agilent G6230 TOF MS	**User Name**	KIB
**Acq Method**	ESI.m	**Acquired Time**	11/24/2022 4:10:23 PM
**IRM Calibration Status**	Success	**DA Method**	ESI.m
**Comment**			

Sample Group **Info.**

Acquisition SW Version 6200 series TOF/6500 series Q-TOF B.05.01 (B5125.2)

User Spectra

Fragmentor Voltage	Collision Energy	Ionization Mode
400	0	ESI

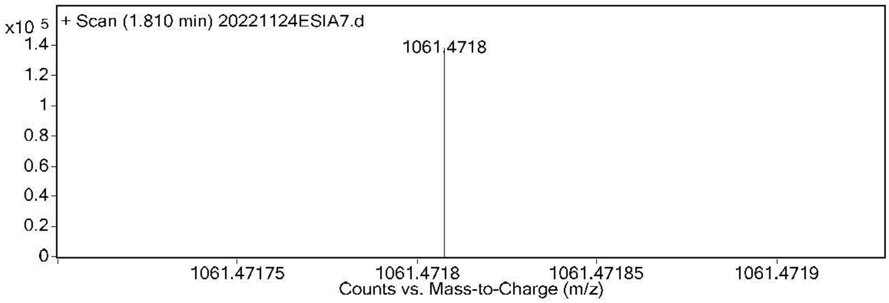

Peak List

m/z	z	Abund	Formula	Ion
857.4657	1	33723.2		
922.0098	1	48368.4		
1019.5185	1	54652.85		
1020.5211	1	28420.66		
1027.4868	1	36759.5		
1028.4885	1	20096.37		
1061.4718	1	137733.5	C55 H74 Na O19	M+
1062.4747	1	78992.97	C55 H74 Na O19	M+
1063.477	1	23792.72	C55 H74 Na O19	M+
1181.5688	1	20248		

Formula Calculator Element Limits

Element	Min	Max
C	0	200
H	0	400
O	15	23
Na	1	1

Formula Calculator Results

Formula	CalculatedMass	Mz	Diff.(mDa)	Diff. (ppm)	DBE
C55 H74 Na O19	1061.4722	1061.4718	0.4	0.4	18.5

--- End Of Report ---

Figure S43 HRESIMS spectrum of compound **7**

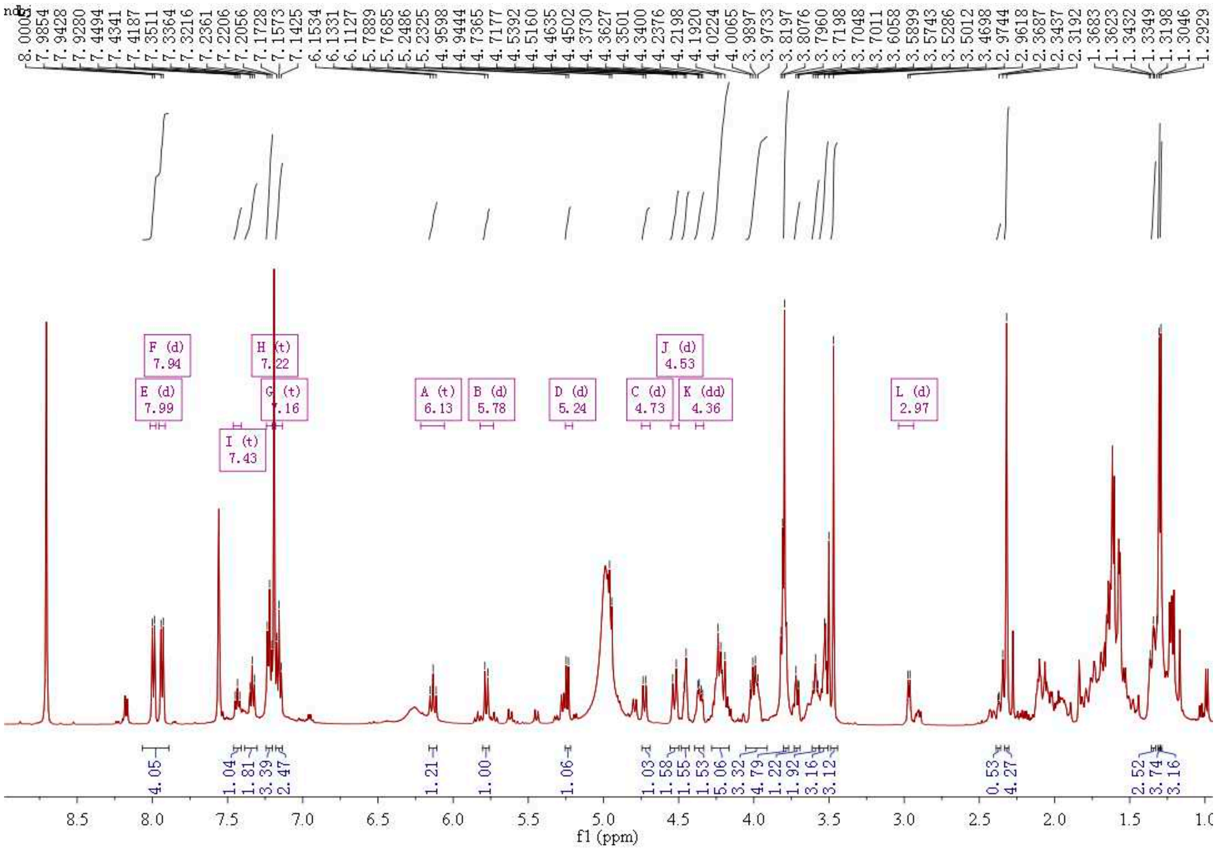

Figure S44 ^{1}H-NMR (500 MHz, pyridine-d_5) spectrum of compound **7**

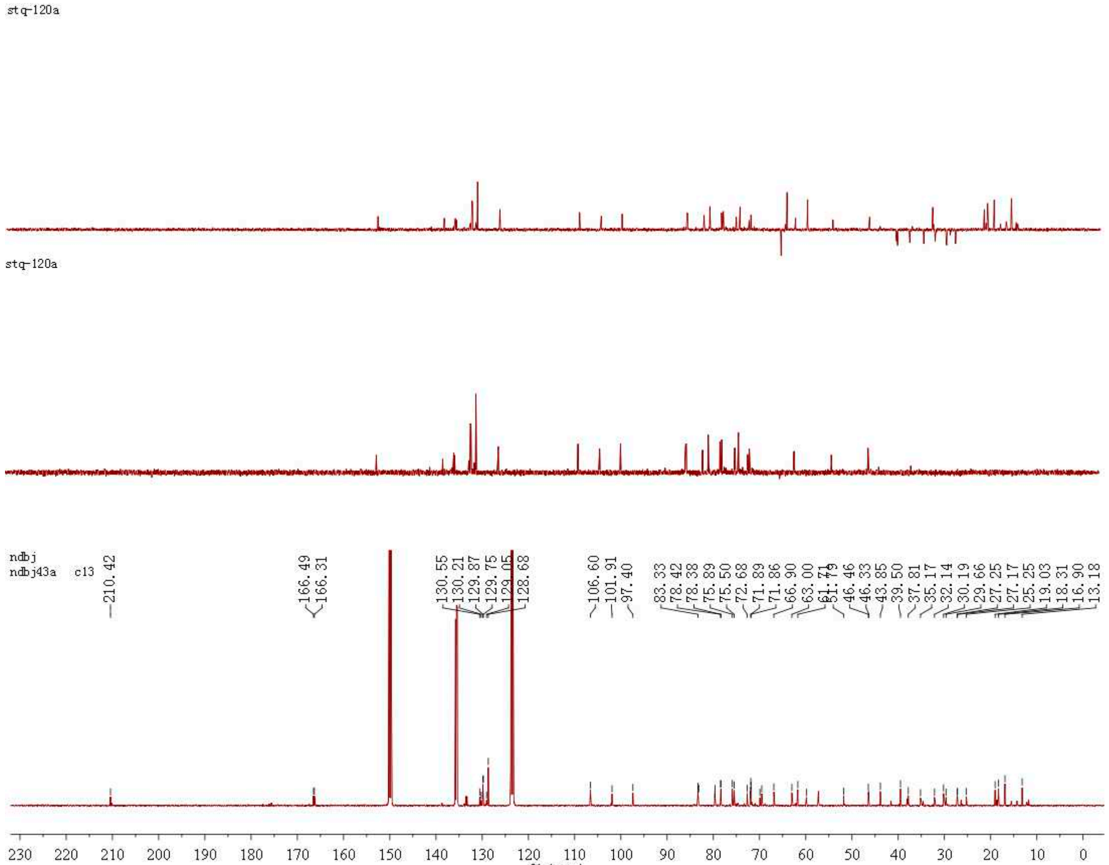

Figure S45 ^{13}C-NMR and DEPT (125 MHz, pyridine-d_5) spectrum of compound **7**

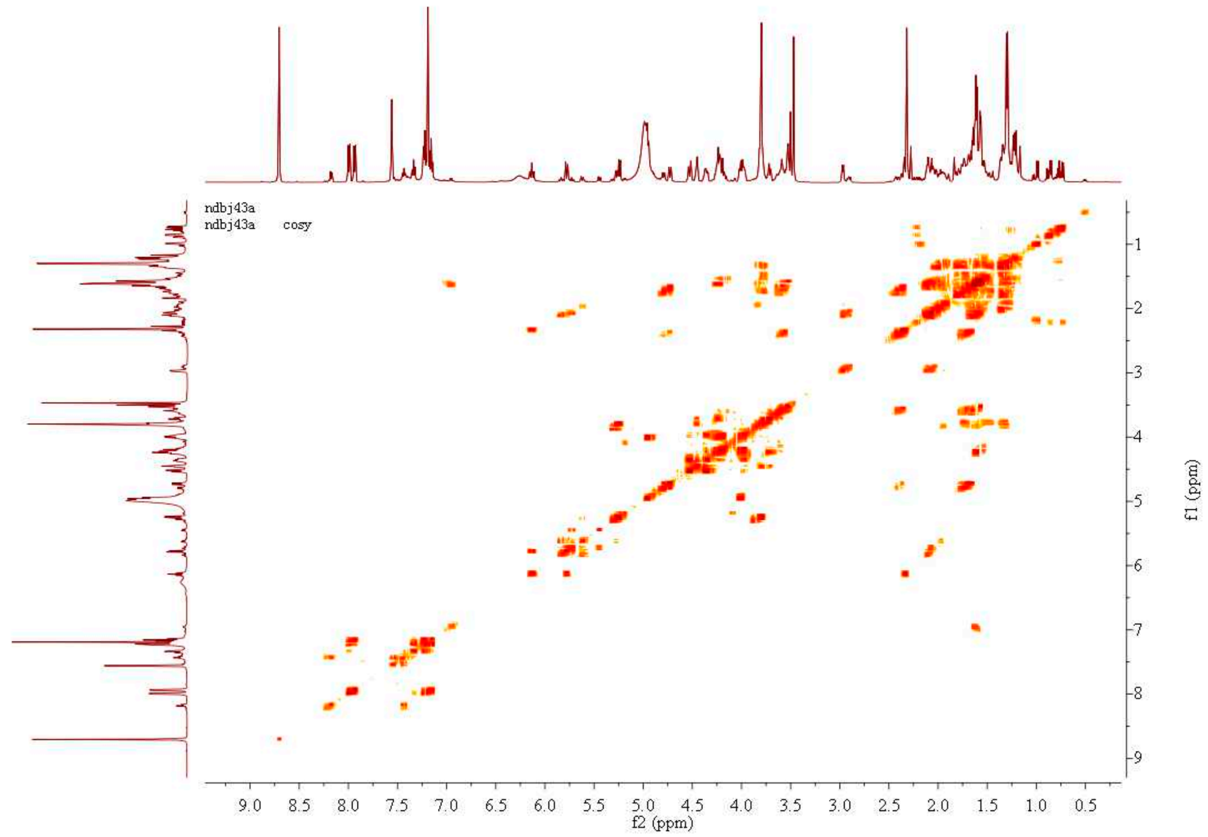

Figure S46 ^{1}H-^{1}H COSY spectrum of compound **7**

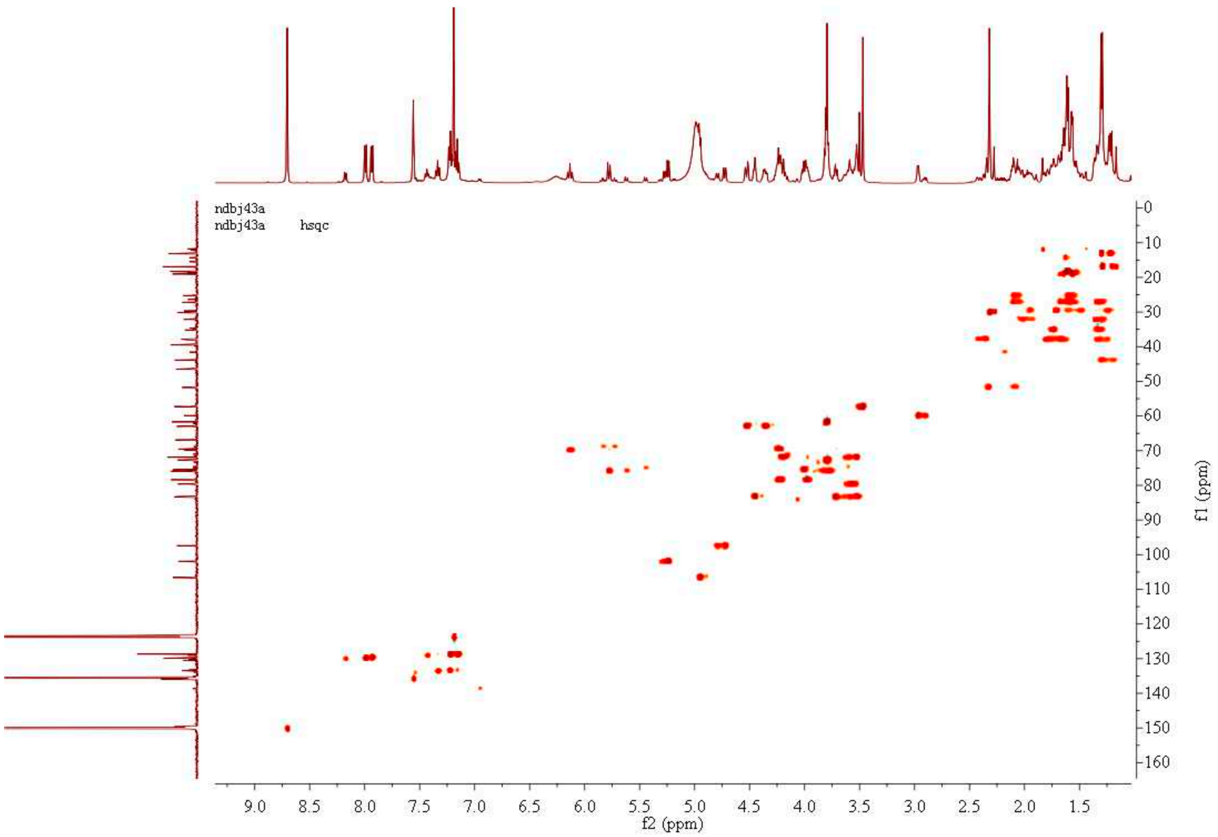

Figure S47 HSQC spectrum of compound **7**

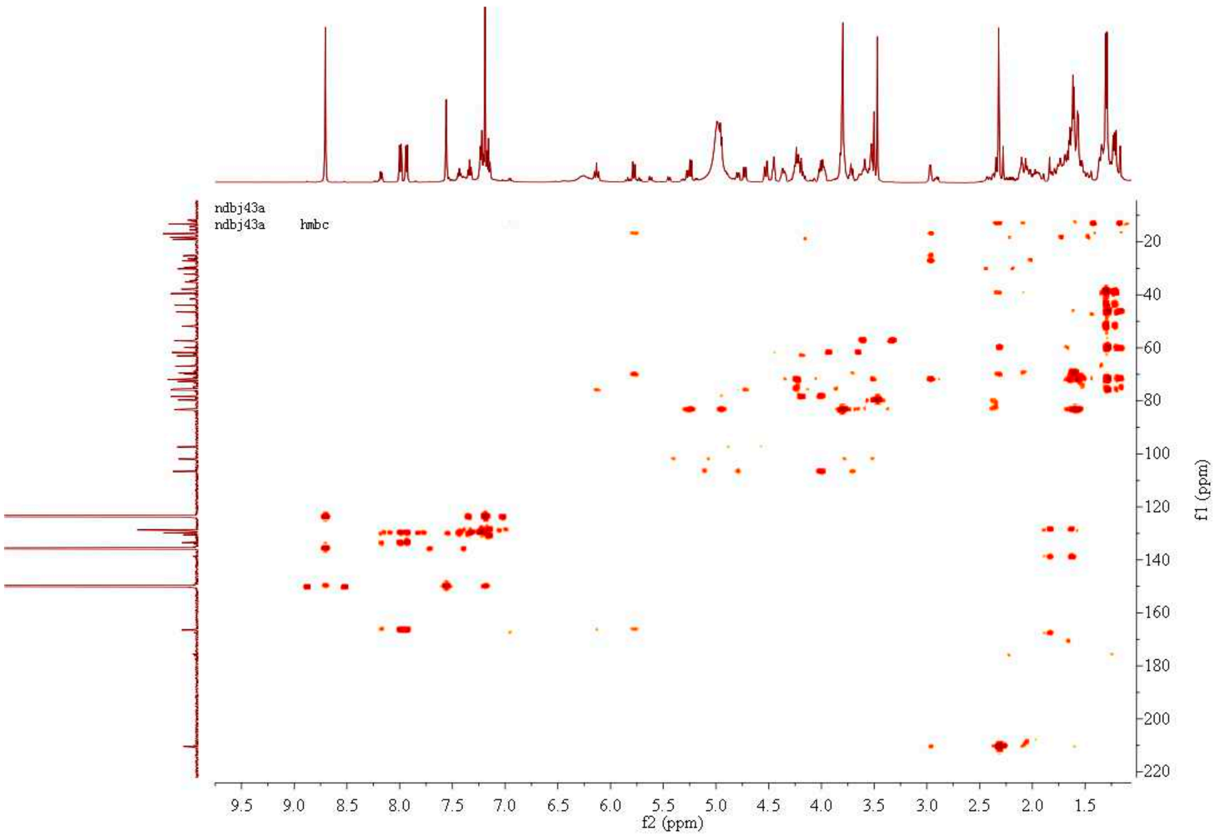

Figure S48 HMBC spectrum of compound **7**

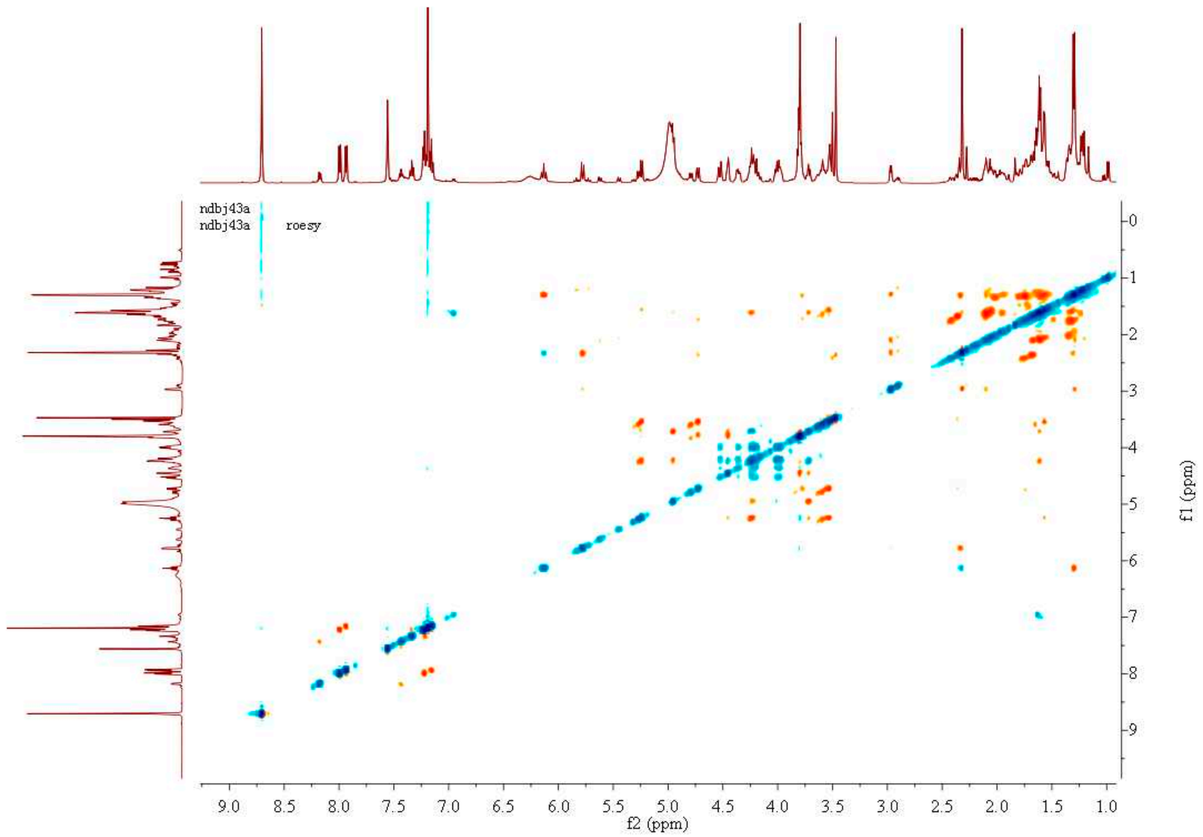

Figure S49 ROESY spectrum of compound **7**

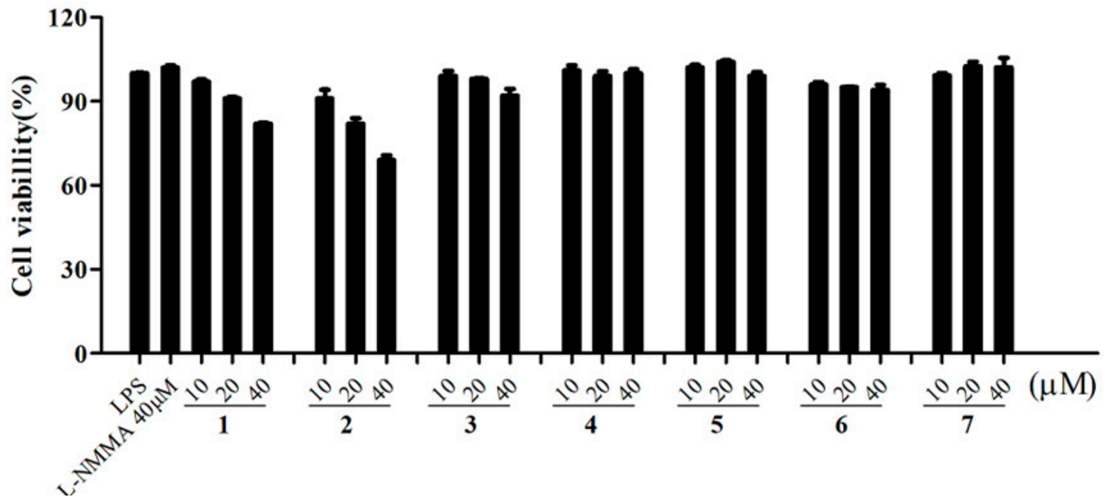

Figure S50 Effects of compounds **1**–**7** on cell viability.
